# Skin commensal bacterium *Staphylococcus epidermidis* CCSM0287 cell-free supernatant protects HaCaT cells from UVB irradiation-induced photodamage and improves skin barrier function

**DOI:** 10.1128/spectrum.03649-25

**Published:** 2026-09-03

**Authors:** Gang Liu, Yan Li, Suzhen Yang, Hong Jiang, Laiji Ma, Qingwen Jia, Tianming Bai, Li Shao

**Affiliations:** 1 Faculty of Flavour Fragrance and Cosmetics, Shanghai Institute of Technology 92263, Shanghai, China; 2 R&D Innovation Center, Shandong Freda Biotech Co., Ltd., Jinan, China; University of Mississippi, University, Mississippi, USA

**Keywords:** *Staphylococcus epidermidis*, UVB, HaCaT cell, antioxidation, anti-inflammatory, skin barrier

## Abstract

Ultraviolet B (UVB) radiation is a major environmental stressor that induces oxidative stress, inflammation, and skin barrier dysfunction. Although *Staphylococcus epidermidis* (*S. epidermidis*) is a beneficial skin commensal, current evidence on its protective effects against UVB-induced skin damage has primarily focused on reference strains or a limited number of individual metabolites. The broader photoprotective potential of postbiotics derived from newly isolated skin commensal strains remains insufficiently characterized. Here, we investigated the anti-photodamage activity and underlying mechanisms of the cell-free supernatant of *S. epidermidis* CCSM0287 (SE 287-CFS), a multifunctional skin commensal isolated from healthy skin, in UVB-induced HaCaT cells. SE 287-CFS significantly reduced intracellular reactive oxygen species (ROS), malondialdehyde (MDA), and pro-inflammatory cytokines, while enhancing antioxidant enzyme activity and interleukin-10 (IL-10) secretion. Mechanistically, SE 287-CFS inhibited p65 nuclear translocation and suppressed activation of the ROS/MAPK/NF-κB signaling pathway. In addition, SE 287-CFS increased transepithelial electrical resistance (TEER) and promoted keratinocyte proliferation and migration, indicating improved skin barrier function. Non-targeted metabolomics combined with correlation analysis suggested that butyric and adipic acids were primarily associated with antioxidative and anti-inflammatory effects. Other organic acids, including succinic, acetic, and propanoic acids, may contribute to skin barrier repair. Together, this study demonstrates that SE 287-CFS protects against UVB-induced photodamage by modulating oxidative stress, suppressing inflammation, and improving skin barrier function. It also highlights the potential of postbiotics derived from skin commensals as candidates for microbiome-based skin photoprotection.

**IMPORTANCE:**

*Staphylococcus epidermidis* is a dominant commensal skin bacterium that plays a crucial role in maintaining skin homeostasis and defending against various external stressors. UV radiation is a primary environmental factor that induces oxidative stress and inflammatory responses, resulting in photodamage and impaired skin barrier function. This study demonstrated the significant protective effect of SE 287-CFS in counteracting ultraviolet B (UVB)-induced skin damage. By modulating the reactive oxygen species (ROS)/MAPK/NF-κB signaling pathways, SE 287-CFS effectively alleviated oxidative stress, reduced inflammatory cytokine production, and enhanced skin barrier function. This research highlighted the potential of using skin commensal bacteria as a novel therapeutic strategy to enhance skin protection against UVB-induced damage, providing a promising approach to improve skin health under environmental stressors like UV radiation.

## INTRODUCTION

The skin acts as a physical, chemical, and immune barrier against the external environment, serving as our first line of defense against toxins, ultraviolet (UV) radiation, and pathogenic insults (1). The skin also serves as an interface to the external environment and harbors a diverse collection of microorganisms, including bacteria, viruses, fungi, and archaea, which interact dynamically with the host (2). Commensal skin microorganisms occupy various skin niches and play a crucial role in maintaining cutaneous barrier function, skin balance, and health, protecting the skin from pathogenic or harmful organisms (3). A deeper understanding of skin microorganisms is essential for developing novel approaches to skin disease treatment. *Staphylococcus epidermidis*, a commensal bacterium, is one of the most abundant bacterial colonizers of healthy human skin and is generally considered beneficial for the skin barrier (4). Numerous studies have shown that *S. epidermidis* is a guardian of skin health, promoting skin immunity, repair, and antimicrobial defense (5–8). In addition to modulating the innate immune response to skin infections or damage, *S. epidermidis* colonization contributes to the development and priming of the adaptive immune system, enhancing both innate and adaptive defenses against pathogens and promoting homeostasis (9).

UV radiation is a major environmental stressor on the skin, with ultraviolet B (UVB) constituting about 5% of the total ultraviolet radiation. Despite its small proportion, UVB is the most potent in damaging the epidermis and dermis, leading to sunburn, melanin formation, collagen loss, and the development of inflammatory skin diseases, photoaging, immunosuppression, and skin cancer (10). The depletion of the atmospheric ozone layer has led to increased UV radiation, significantly boosting the demand for ultraviolet protection products (11). Therefore, it is urgently needed to explore novel bioactive compounds, such as plant extracts (12), probiotics (13), postbiotics (14), and bacterial metabolites (15), to prevent UV-induced photoaging and damage. Oxidative stress and inflammation are key molecular events in UVB-induced skin injury, characterized by excessive production of reactive oxygen species (ROS) and the release of pro-inflammatory cytokines (16, 17). These processes further promote cellular damage, induce senescence, and impair skin barrier function (18). Because UVB-induced skin injury involves multiple interconnected pathogenic mechanisms, natural bioactive substances that can modulate different aspects of skin damage have attracted increasing attention. Many research studies have indicated that the skin microbiome modulates the dermal response to UV irradiation (19). Skin commensal *S. epidermidis* exhibits probiotic-like properties and plays a beneficial role in maintaining skin health (20). Therefore, bioactive substances derived from skin microorganisms may represent a promising source of photoprotective agents. However, current evidence on the protective effects of skin commensal microorganisms against UVB-induced skin damage remains limited. A study has shown that the skin microbiome provides immune protection by orchestrating local cellular and innate immune responses to UVB (21). *S. epidermidis* ATCC 12228 and its by-product lipoteichoic acid have been reported to promote melanocyte survival and proliferation under UVB-induced DNA damage. This effect is associated with the upregulation of TRAF1, CASP14, CASP5, and TP73 (22). A previous study demonstrated that butyric acid produced by the skin probiotic *S. epidermidis* ATCC 12228 down-regulated UVB-induced pro-inflammatory cytokine production via free fatty acid receptor 2 (23). Balasubramaniam et al. (24) reported that glycerol fermentation by *S. epidermidis* ATCC 12228 generates electricity, which inhibited UVB-induced labile iron accumulation in the skin and regulated iron redox cycling. To date, evidence on the protective effects of *S. epidermidis* against UVB-induced skin injury has primarily focused on the reference strain ATCC 12228 and its fermentation-derived products. However, the capacity of *S. epidermidis*-derived postbiotics to alleviate UVB-induced oxidative stress, inflammation, and skin barrier dysfunction has not been systematically characterized. Moreover, the relationships between specific metabolites in *S. epidermidis*-derived postbiotics and their beneficial effects remain poorly understood. Therefore, the identification and characterization of novel *S. epidermidis* strains isolated from healthy skin may facilitate the exploration of skin probiotic resources and improve our understanding of metabolite-function relationships.

In our previous studies, more than 200 strains of *S. epidermidis* were isolated from healthy skin at various anatomical sites. The antioxidant and anti-inflammatory potential of their cell-free supernatants was then preliminarily evaluated using the DPPH radical scavenging activity and hyaluronidase inhibition *in vitro* (25–27). Among these isolates, *S. epidermidis* CCSM0287 exhibited particularly strong biofunctional activity (China Patent: ZL202210704624.6). Based on these observations, this study aimed to investigate the protective effects of SE 287-CFS against UVB-induced photodamage. Specifically, we evaluated its ability to alleviate oxidative stress, suppress inflammation, and improve skin barrier function. In addition, non-targeted metabolomics combined with correlation analysis was used to identify candidate metabolites associated with the observed protective effects. By linking strain-specific functional phenotypes with metabolite profiles, this study may expand current understanding of the functional potential of the skin commensal *S. epidermidis*, provide a framework for screening microbiome-derived postbiotics with photoprotective activity, and support the development of microbiome-based strategies for skin protection and disease intervention. In addition, this integrative approach may facilitate mechanistic studies and aid in identifying candidate bioactive molecules with translational or industrial potential.

## MATERIALS AND METHODS

### Bacterial strain and cell lines

*S. epidermidis* CCSM0287 was isolated, purified, and identified from healthy facial skin and deposited in the China Center for Type Culture Collection under accession number CCTCC M2022779 (28). Unless otherwise noted, the strain was grown in Trypticase Soy Broth (TSB) (Hopebio, HB4114, China) or on Soybean-Casein Digest Agar (Hopebio, HB7026-9, China) plates at 37°C with/without shaking (160 r/min). Immortalized human keratinocyte cells (HaCaT) were purchased from iCell Bioscience Inc. (iCellbioscience, iCell-h066, China). The cells were cultured in DMEM (HyClone, SH30021, UT) containing 4.5 g/L D-glucose supplemented with 10% fetal bovine serum at 37°C in a humidified atmosphere with 5% carbon dioxide. The cells were used for further experiments until they reached 80%–90% confluence in plates.

### Preparation of the cell-free supernatant

A pure culture of *S. epidermidis* CCSM0287 was obtained after three passages and cultured for 10 h in TSB once the strain reached a steady state. SE 287-CFS was then obtained from bacterial cultures by centrifugation at 10,000 × *g*, 4°C for 10 min, followed by sequential filtration through 0.45 µm (Titan, 02036323, China) and 0.22 µm (Titan, 02036322, China) cell strainers. SE 287-CFS was directly added to fresh HaCaT culture medium and thoroughly mixed before being applied to the cells. The concentration of SE 287-CFS was determined based on its volume ratio, with final concentrations (1%, 2%, 4%, 6%, 8%, and 10%) adjusted by varying its proportion in the medium. The activities of superoxide dismutase (SOD) and glutathione peroxidase (GSH-PX) in SE 287-CFS were measured using the SOD assay kit (Nanjing Jiancheng, A001-1, China) and the GSH-PX assay kit (Nanjing Jiancheng, A005-1, China), respectively.

### UVB irradiation

The method was slightly modified according to Li et al. (29). HaCaT cells were seeded in 96-well, 24-well, or 6-well plates at the specified density. Before UVB irradiation, the medium volume was reduced to improve irradiation efficiency. Specifically, 35, 60, and 200 µL of medium were retained in 96-, 24-, and 6-well plates, respectively, while ensuring that the cells remained covered at the bottom of each well. This was done to avoid a large amount of culture medium affecting the irradiation efficiency of UVB. The cells were then exposed to UVB irradiation at doses of 10, 20, 30, and 40 mJ/cm^2^, with corresponding exposure times of 100, 200, 300, and 400 s, and an irradiation intensity of 100 µW/cm^2^. The plate was kept on ice to prevent overheating during the irradiation process. UVB irradiation conditions were established using a UV lamp (PHILIPS, PL-S 9W/01, Poland) with a wavelength of 311 nm. Irradiation intensity was measured using a UV radiation intensity meter (HXMETE, HX8203, China). To determine the optimal UVB irradiation conditions, after UVB irradiation, the medium was replaced with fresh medium. After 24 h of cell culture, the harvested cells were analyzed by cell viability and qPCR to establish a UVB-induced HaCaT cell model. Before treatment with SE 287-CFS or *N*-acetylcysteine (NAC), the cells were irradiated with UVB. Then, SE 287-CFS or NAC was mixed evenly or dissolved in fresh basal culture medium. After that, the cells were treated with the drug by changing the medium and continued to be cultured for the set time. Then, the cells were collected for further analysis.

### Cell viability

HaCaT cells were seeded in 96-well plates at a density of 2 × 10⁴ cells per well and incubated for 16 h. Cell viability was measured using a Cell Counting Kit-8 (CCK-8, Biosharp, BS350b, China) according to the manufacturer’s instructions. Briefly, cells were exposed to UVB and treated with SE 287-CFS, after which CCK-8 solution was then added to each well and incubated for an additional 2 h. Absorbance at 450 nm was measured using a microplate reader (Diatek, DR-200Bs, China). Untreated cells served as the control group, and the medium without cells served as the blank group.

### qPCR assay of mRNA expression of inflammatory cytokines

The mRNA levels of cytokines in treated cells were normalized to GAPDH and determined by qPCR. HaCaT cells were seeded in 6-well plates at a density of 5 × 10^5^ cells per well and incubated for 16 h. Following UVB irradiation at various doses or treatment with 30 mJ/cm^2^ plus 4% SE 287-CFS, total RNA was extracted using TRIpure Total RNA Extraction Reagent (ELK Biotechnology, EP013, China). RNA was then reverse transcribed into cDNA using EntiLink 1st Strand cDNA Synthesis Super Mix (ELK Biotechnology, EQ031, China) according to the manufacturer’s instructions. qPCR analysis was conducted using EnTurbo SYBR Green PCR SuperMix (ELK Biotechnology, EQ001, China) and the QuantStudio 6 Flex System PCR Instrument, following the manufacturer’s instructions. Transcript levels were normalized to GAPDH, which served as an internal control. The primer sequences are provided in Table 1.

**TABLE 1:** The qPCR primer sequences of genes used in the study

Gene name		Primer sequence
Human-tumor necrosis factor-α (TNF-α)	Forward	CTCTTCTCCTTCCTGATCGTGG
	Reverse	CTTGTCACTCGGGGTTCGAG
Human-IL-6	Forward	ACTGGCAGAAAACAACCTGAAC
	Reverse	TTGTACTCATCTGCACAGCTCTG
Human-IL-1β	Forward	ACGATGCACCTGTACGATCACT
	Reverse	GAGAACACCACTTGTTGCTCCA
Human-IL-8	Forward	ACTGAGAGTGATTGAGAGTGGAC
	Reverse	AACCCTCTGCACCCAGTTTTC
Human-cyclooxygenase-2 (COX-2)	Forward	AGATTATGTGCAACACTTGAGTGG
	Reverse	ATTCCTACCACCAGCAACCCT
Human-inducible nitric oxide synthase (iNOS)	Forward	GCAGGACTCACAGCCTTTGG
	Reverse	GGCTGGATGTCGGACTTTGT
Human-IL-10	Forward	AACCTGCCTAACATGCTTCG
	Reverse	GAGTTCACATGCGCCTTGAT
Human-glyceraldehyde-3-phosphate dehydrogenase (GAPDH)	Forward	CATCATCCCTGCCTCTACTGG
	Reverse	GTGGGTGTCGCTGTTGAAGTC

### Drug administration experiment design

Cells were treated with SE 287-CFS at concentrations of 1%, 2%, 4%, 6%, 8%, and 10% (vol/vol) for 24 h, and with NAC at concentrations of 10, 20, and 30 µmol/L for 3 h. Thirty-five microliters of medium was reserved for UVB irradiation at a dose of 30 mJ/cm^2^, which was used to determine the optimal SE 287-CFS and NAC concentrations. Cells were then treated with 4% (vol/vol) SE 287-CFS for 4, 8, 12, 16, 18, and 20 h. The optimal treatment time for SE 287-CFS was determined under the 30 mJ/cm^2^ UVB irradiation condition. The ability of SE 287-CFS to restore UVB-induced damage in HaCaT cells was assessed using the Cell Counting Kit-8 method. The production and expression of pro-inflammatory and anti-inflammatory cytokines in SE 287-CFS-treated HaCaT cells exposed to UVB irradiation were measured using ELISA kits. Cells were stimulated with 4% SE 287-CFS for 18 h. The supernatant was collected to measure the content of TNF-α (ELK Biotechnology, ELK1190, China), IL-1β (ELK Biotechnology, ELK1270, China), IL-6 (ELK Biotechnology, ELK1156, China), IL-8 (ELK Biotechnology, ELK1159, China), COX-2 (ELK Biotechnology, ELK5230, China), iNOS (ELK Biotechnology, ELK2643, China), and interleukin-10 (IL-10) (ELK Biotechnology, ELK1142, China) using ELISA kits, following the standard instructions.

### Measurement of intracellular ROS production

The effects of SE 287-CFS on intracellular ROS levels in UVB-induced HaCaT cells were measured using 2′,7′-dichlorodihydrofluorescein diacetate (DCFH-DA) (Beyotime, S0033, China) as a fluorescent probe, according to the manufacturer’s instructions. Briefly, the UVB-induced HaCaT cell model was treated with 4% SE 287-CFS for 18 h, followed by staining with 10 μM DCFH-DA in PBS for 30 min at 37°C. The cells were washed three times with PBS. Next, Hoechst (Solarbio, B8040, China)-stained nuclei were added dropwise and incubated at room temperature, protected from light, for 30 min. The cells were then washed three times with serum-free medium. The cells were imaged using a fluorescence microscope (Nikon, Eclipse Ci-L, Japan) immediately after staining was completed.

### Detection of MDA, nitric oxide, SOD, catalase, and glutathione levels

HaCaT cells (5 × 10^5^ cells per well) were seeded in 6-well plates. After treatment with 30 mJ/cm^2^ UVB, 4% SE 287-CFS, and 20 µmol/L NAC, malondialdehyde (MDA) was detected using the TBA method and the MDA assay kit (Nanjing Jiancheng, A003-1, China). The cellular nitric oxide (NO) content was measured using the NO assay kit (Nanjing Jiancheng, A013-2-1, China), following the standard instructions. The levels of antioxidants, including SOD, catalase (CAT), and intracellular reduced GSH, were assessed separately using the T-SOD assay kit (Nanjing Jiancheng, A001-1, China), the CAT assay kit (Nanjing Jiancheng, A007-1-1, China), and the GSH assay kit (Nanjing Jiancheng, A006-2-1, China), following the instructions.

### Western blot analysis

HaCaT cells (5 × 10^5^ cells per well) were seeded into 6-well plates. Cells were treated with 4% SE 287-CFS for 18 h, followed by UVB stimulation at 30 mJ/cm^2^. The cells were lysed on ice with ice-cold RIPA lysis buffer (ASPEN, AS1004, China), containing a protease cocktail and phosphatase inhibitors, for 30 min. The lysate was then centrifuged at 12,000 × *g* for 5 min at 4°C to collect the supernatant. Protein concentration was determined using a BCA kit (ASPEN, AS1086, China). Forty micrograms of cytoplasmic or nuclear protein samples were boiled with loading buffer for 5 min, followed by separation on a 12% SDS-PAGE gel and transfer to 0.45 µm PVDF membranes (Millipore, IPVH00010, USA). The membrane was blocked with 5% skim milk for 1 h, then incubated with primary antibodies at 4°C overnight, following the manufacturer’s instructions and the appropriate dilution ratio. After three washes with 1× Tris-buffered saline containing 0.1% Tween-20 (5 min per wash), the membrane was incubated with HRP-conjugated goat anti-rabbit IgG for 30 min. The antibody-specific protein was visualized using an enhanced chemiluminescence detection system with an ECL kit (ASPEN, AS1059, China), and the gray value of the target zone was analyzed using the AlphaEaseFC software.

### Immunofluorescence assay of NF-κB nuclear translocation

HaCaT cells (5 × 10^5^ cells per well) were seeded into 6-well plates. Cells were treated with 4% SE 287-CFS for 18 h, followed by UVB stimulation at 30 mJ/cm^2^. To analyze the nuclear translocation of NF-κB, cells were first washed with PBS to remove the culture medium, then fixed with 4% paraformaldehyde for 20 min. The primary NF-κB p65 antibody was diluted 1:200 with 5% BSA, and the cells were incubated with the antibody overnight at 4°C. After returning to room temperature, the cells were incubated with CoraLite488-conjugated Goat Anti-Rabbit IgG secondary antibody at 37°C for 40 min, protected from light. The cells were washed three times with PBS (5 min per wash) after each step. Finally, DAPI was used as a nuclear stain and incubated with HaCaT cells at room temperature for 30 min, protected from light. Images were then captured using a confocal laser scanning microscopy (Carl Zeiss, ZEISS-LSM880, Germany).

### Detection of TEER

HaCaT cells were seeded at a density of 1 × 10^5^ cells per well on a polycarbonate 24-well transwell membrane (Corning, 3422, USA) with a mean pore size of 8.0 μm and a surface area of 0.33 cm^2^. The cells were treated with 30 mJ/cm^2^ UVB or 30 mJ/cm^2^ UVB plus SE 287-CFS. After 18 h, the resistance value was recorded using an Electrical Resistance System (Millicell, ERS2, USA). Simultaneously, the resistance of the cell-free group (blank group) was also measured. Transepithelial electrical resistance (TEER) was calculated using the following formula: TEER (Ω·cm^2^) = (Measured resistance − Blank resistance)/Monolayer membrane area (0.33 cm^2^).

### Cell migration

Cell migration was assessed using the cell scratch assay. Briefly, HaCaT cells were seeded into a 6-well plate at a density of 5 × 10⁵ cells per well and incubated until a confluent monolayer was formed. A scratch wound was then made using a sterile 200 μL pipette tip to create a line across the bottom of the culture dish. The scratched cells were then treated with either 30 mJ/cm^2^ UVB or 30 mJ/cm^2^ UVB plus 4% SE 287-CFS. The scratched cells were gently rinsed with PBS, and images of the scratches were taken at the 0 h time point under a microscope. Incubation continued, and the wound area was imaged using a microscope (OLYMPUS, IX51, Japan) at the 10 and 18 h time points. The migration area was then quantified using an imaging system (Q-IMAGING, MicroPublisher, CAN) to assess the rate of wound closure. The experiment included three independent biological replicates. The cell migration rate was calculated using the following formula: Cell migration rate (%) = [(Initial scratch width − Final scratch width)/Initial scratch width] × 100.

### EdU staining method

HaCaT cells (5 × 10^5^ cells per well) were seeded into 6-well plates. Cells were treated with 4% SE 287-CFS for 18 h, followed by UVB stimulation at 30 mJ/cm^2^. The medium was removed, and the cells were fixed with 4% paraformaldehyde for 20 min. The membrane-breaking solution was prepared and incubated at room temperature for 10 min. Subsequently, 1× EdU-488 green light staining solution was prepared and incubated at 37°C for 30 min, protected from light. The cells were washed three times with PBS (5 min per wash) at the end of each step. Finally, 1× Hoechst 33342 (Beyotime, C1022, China) was added dropwise for nuclear staining. The cells were incubated for 30 min at room temperature, protected from light, washed with PBS, and photographed under a microscope (Eclipse Ci-L).

### Non-targeted metabolomics analysis

For metabolite extraction, 200 μL of SE 287-CFS or TSB was mixed with 800 μL of precooled methanol–acetonitrile (1:1) solution. The mixture was vortexed for 30 s to ensure thorough mixing. Ultrasonic extraction was then performed at 5°C for 30 min at a frequency of 40 kHz. The samples were allowed to stand at −20°C for 30 min, followed by centrifugation at 13,000 × *g* for 15 min at 4°C. The supernatant was removed, and the samples were dried under a stream of nitrogen. The concentrated samples were dissolved in 120 μL of acetonitrile:water (1:1) solution and vortexed for 30 s. Low-temperature ultrasonic extraction was then conducted at 5°C for 5 min at a frequency of 40 kHz. Finally, the samples were centrifuged at 13,000 × *g* for 10 min at 4°C, and the supernatant was transferred for LC-MS analysis. Quality control (QC) samples were prepared by combining equal volumes of extract from each sample. The volume of the QC sample was equal to that of the corresponding analytical sample, and both were treated and analyzed using identical methods to assess the stability of the testing process. LC-MS analysis was performed using a Q-Exactive HF-X mass spectrometer (Thermo Scientific, USA) coupled with a Vanquish Horizon system (Thermo Scientific, USA). Chromatographic conditions were as follows: an ACQUITY UPLC HSS T3 column (100 mm × 2.1 mm, 1.8 µm, Waters, Milford, USA) was used. Mobile phase A consisted of 95% water and 5% acetonitrile (containing 0.1% formic acid), mobile phase B contained 47.5% acetonitrile, 47.5% isopropanol, and 5% water (containing 0.1% formic acid), the injection volume was 3 μL, and the column temperature was maintained at 40°C. Mass spectrometry conditions were as follows: the scan range was 70 to 1,050 *m*/*z*, sheath gas flow rate was 50 arb, auxiliary gas flow rate was 13 arb, heater temperature was 425°C, and capillary temperature was 325°C. The spray voltage (+) was 3,500 V, and the spray voltage (−) was −3,500 V. The S-Lens RF level was 50, normalized collision energies were 20%, 40%, and 60%. The resolution for full MS was 60,000, and for MS^2^ was 7,500. This metabolomics study was conducted in the laboratory of Shanghai Majorbio Bio-pharm Technology Co., Ltd. The Majorbio Cloud platform (https://cloud.majorbio.com) was used for analysis.

### Statistical analysis

Data are expressed as mean ± SD. Statistical analysis was conducted using one-way ANOVA (GraphPad Prism 9.5), followed by Tukey’s multiple comparisons test to correct for multiple comparisons and adjust *P* values. In the one-way ANOVA, group differences were assessed, with significance levels marked as (*) *P* < 0.05, (**) *P* < 0.01, (***) *P* < 0.001, and (****) *P* < 0.0001. Graphs were generated using GraphPad. Pearson correlation analysis was conducted using R version 4.5.0 and the cor.test function to assess the relationships between oxidative stress, inflammatory markers, and organic acids. The correlation coefficient (r) and its associated *P* value were reported. *P* value < 0.05 was considered statistically significant. Correlation heatmaps were generated using ChiPlot (https://www.chiplot.online/), an online bioinformatics tool offering visualization options and educational resources. Heatmap was plotted by https://www.bioinformatics.com.cn.

## RESULTS

### Effect of SE 287-CFS on UVB-induced inflammatory damage

**Fig 1:**
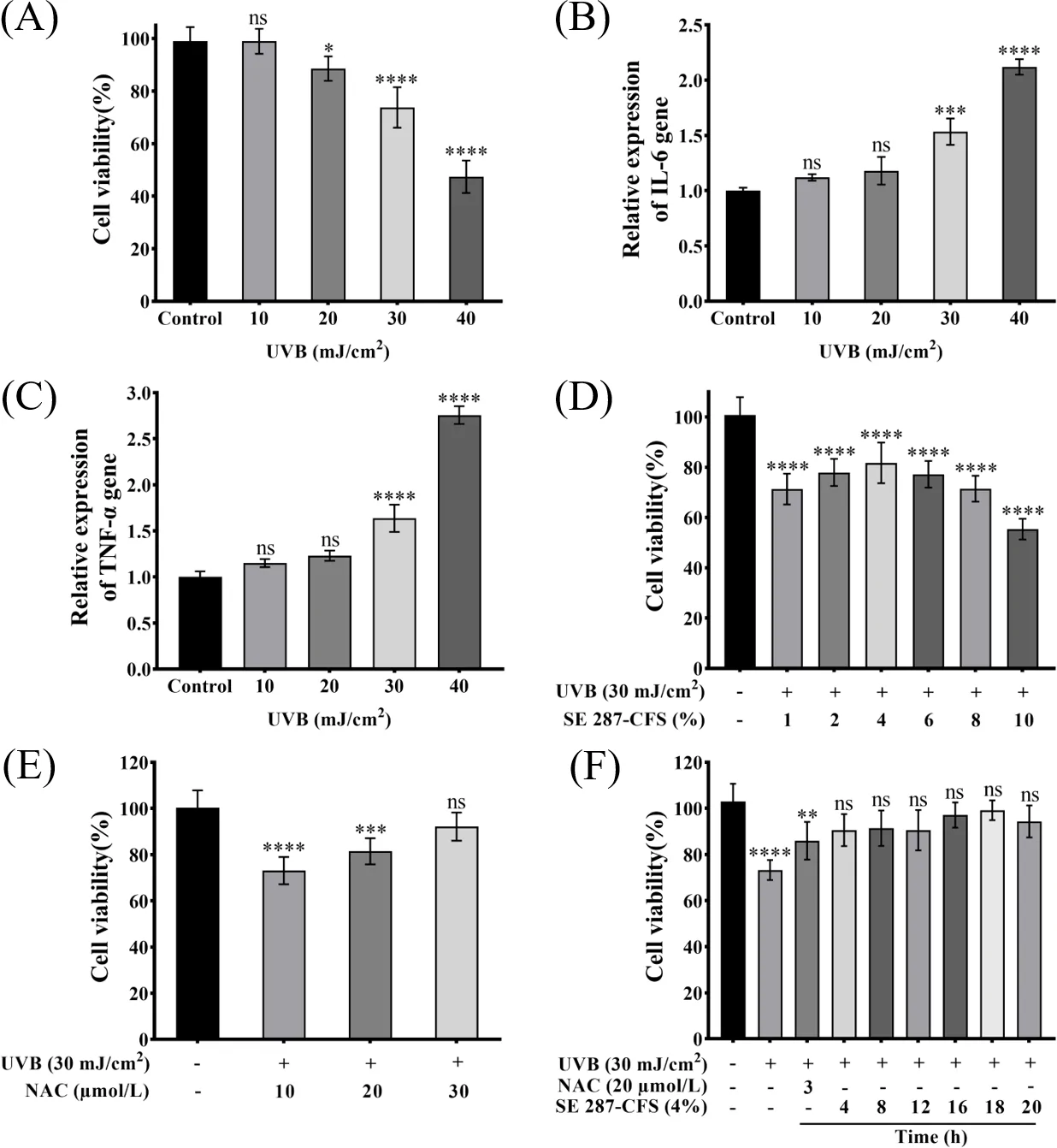
Establishment of a UVB-induced HaCaT cell inflammation model and the effect of SE 287-CFS on cell viability in the inflammation model. (**A**) Effect of different irradiation doses of UVB on cell viability. (**B**) Effect on UVB-induced IL-6 gene expression measured by qPCR. (**C**) Effect on UVB-induced TNF-α gene expression measured by qPCR. (**D**) Effect of different concentrations of SE 287-CFS on cell viability at a 30 mJ/cm^2^ UVB irradiation dose. (**E**) Effect of different concentrations of NAC on cell viability at a 30 mJ/cm^2^ UVB irradiation dose. (**F**) Effect of different treatment times of 4% concentration of SE 287-CFS on cell viability at a 30 mJ/cm^2^ UVB irradiation dose. Treatment conditions are denoted as “+” (present) or “–” (absent) for UVB, NAC, and SE 287-CFS. The control group consisted of cells without any treatment (“–” for UVB, NAC, and SE 287-CFS), cultured under standard conditions. Cell viability is expressed as mean ± standard deviation (SD) from six biological replicates. Gene expression levels of IL-6 and TNF-α are reported as mean ± SD from three biological replicates. *P < 0.05, **P < 0.01, ***P < 0.001, ****P < 0.0001 vs control group as determined by one-way ANOVA. ns indicated no significant difference.

First, a UVB-induced inflammation model using HaCaT cells was established. The optimal UVB irradiation dose was initially evaluated in this experiment. HaCaT cells were irradiated with UVB at doses of 0, 10, 20, 30, and 40 mJ/cm^2^. Cell viability and the gene expression of TNF-α and IL-6 in HaCaT cells indicated that UVB induces inflammatory responses and cell damage within this irradiation dose range. As shown in Fig. 1A through C, UVB increases the expression of pro-inflammatory cytokine genes and decreases cell viability in HaCaT cells. Compared to the control group (cells without UVB exposure or any treatment), cell viability significantly decreased (*P* < 0.0001) at an irradiation dose of 30 mJ/cm^2^, and IL-6 (*P* = 0.0001) and TNF-α (*P* < 0.0001) gene expression levels were significantly elevated at 30 mJ/cm^2^. Therefore, 30 mJ/cm^2^ was chosen as the optimal irradiation dose for the UVB-induced inflammation model.

The optimal effect concentration and times of SE 287-CFS were also evaluated in this experiment. As shown in Fig. 1D, we evaluated the ability of SE 287-CFS to restore cell viability under 30 mJ/cm^2^ UVB irradiation. The results showed that under the UVB-induced inflammation model, cell viability increased and then decreased with increasing SE 287-CFS concentration, with the highest viability (81.73% ± 8.10%) observed at 4% SE 287-CFS concentration. Therefore, a 4% concentration of SE 287-CFS was selected as the optimal concentration for subsequent experiments. Zhang et al. (30) showed that NAC acts as both an intracellular ROS scavenger and an inhibitor of cellular damage induced during inflammation. The experimental results confirmed that NAC treatment restored cellular damage induced by UVB irradiation (Fig. 1E), and thus, 20 µmol/L NAC was selected as the positive control for subsequent experiments. Finally, we evaluated the optimal treatment time for 4% SE 287-CFS using a cell viability assay. As shown in Fig. 1F, compared to the control group, cell viability increased with treatment time, reaching the highest level (99.18% ± 4.28%) at 18 h. Therefore, 18 h was selected as the optimal treatment time for 4% SE 287-CFS in subsequent experiments.

### Effect of SE 287-CFS on UVB-induced ROS production

**Fig 2:**
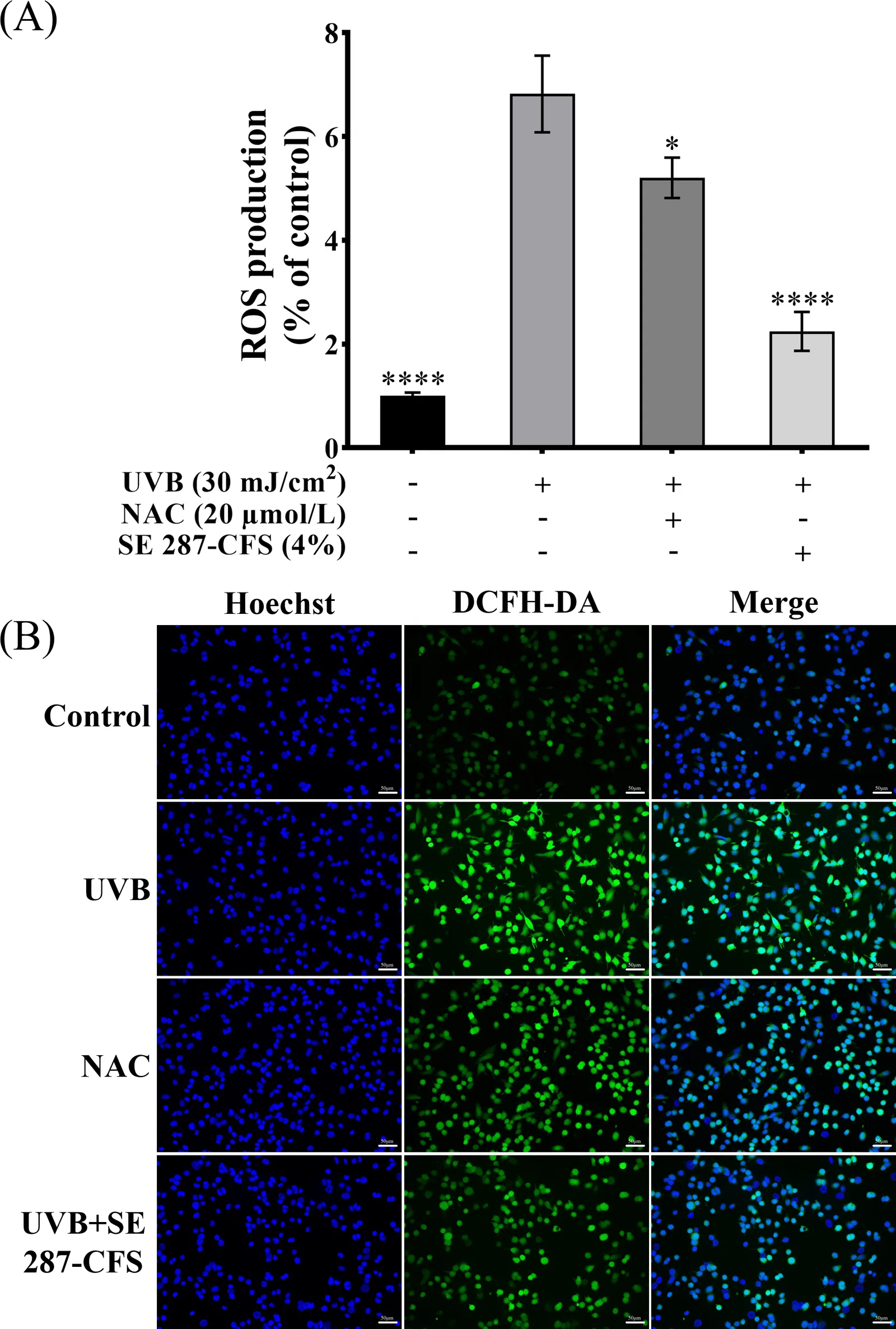
Changes of intracellular ROS in HaCaT cells after UVB irradiation by SE 287-CFS and 20 µmol/L NAC. (**A**) Comparison of ROS fluorescence intensity in HaCaT inflammatory cells of different treatment groups. (**B**) Fluorescence image of ROS content in a 4% concentration of SE 287-CFS treated UVB-induced HaCaT cell model measured by DCFH-DA probe (bars = 50 µm). The UVB group comprised cells exposed to UVB irradiation (“+” for UVB) without additional treatment (“–” for NAC and SE 287-CFS), serving as the UVB-induced damage model. The NAC group comprised cells exposed to UVB (“+” for UVB) and treated exclusively with NAC (“+” for NAC and “–” for SE 287-CFS). The SE 287-CFS group comprised cells exposed to UVB (“+” for UVB) and treated exclusively with SE 287-CFS (“+” for SE 287-CFS and “–” for NAC). Data are expressed as mean ± SD from three biological replicates. *P < 0.05, ****P < 0.0001 vs 30 mJ/cm^2^ UVB group as determined by one-way ANOVA.

As shown in Fig. 2A, ROS levels in the control group (cells without UVB exposure or any treatment) were normalized to a value of 1 as a reference. ROS levels in the UVB group (cells exposed to UVB irradiation but receiving no further treatment), NAC group (cells exposed to UVB irradiation and treated only with NAC), and SE 287-CFS group (cells exposed to UVB irradiation and treated only with SE 287-CFS) were expressed as relative ratios compared to this reference. After UVB irradiation, intracellular ROS levels in the UVB group increased 5.82-fold compared to the control group, with a highly significant difference (*P* < 0.0001). In the UVB-induced HaCaT cell model, both 20 µmol/L NAC and 4% SE 287-CFS exhibited protective effects, reducing ROS levels by 23.73% (*P* = 0.011) and 67.08% (*P* < 0.0001), respectively, compared to the UVB group. These results suggest that SE 287-CFS is more effective in eliminating UVB-induced ROS compared to 20 µmol/L NAC. Fluorescence images also confirm this phenomenon (Fig. 2B).

### Effect of SE 287-CFS on intracellular MDA, SOD, CAT, and GSH production

**Fig 3:**
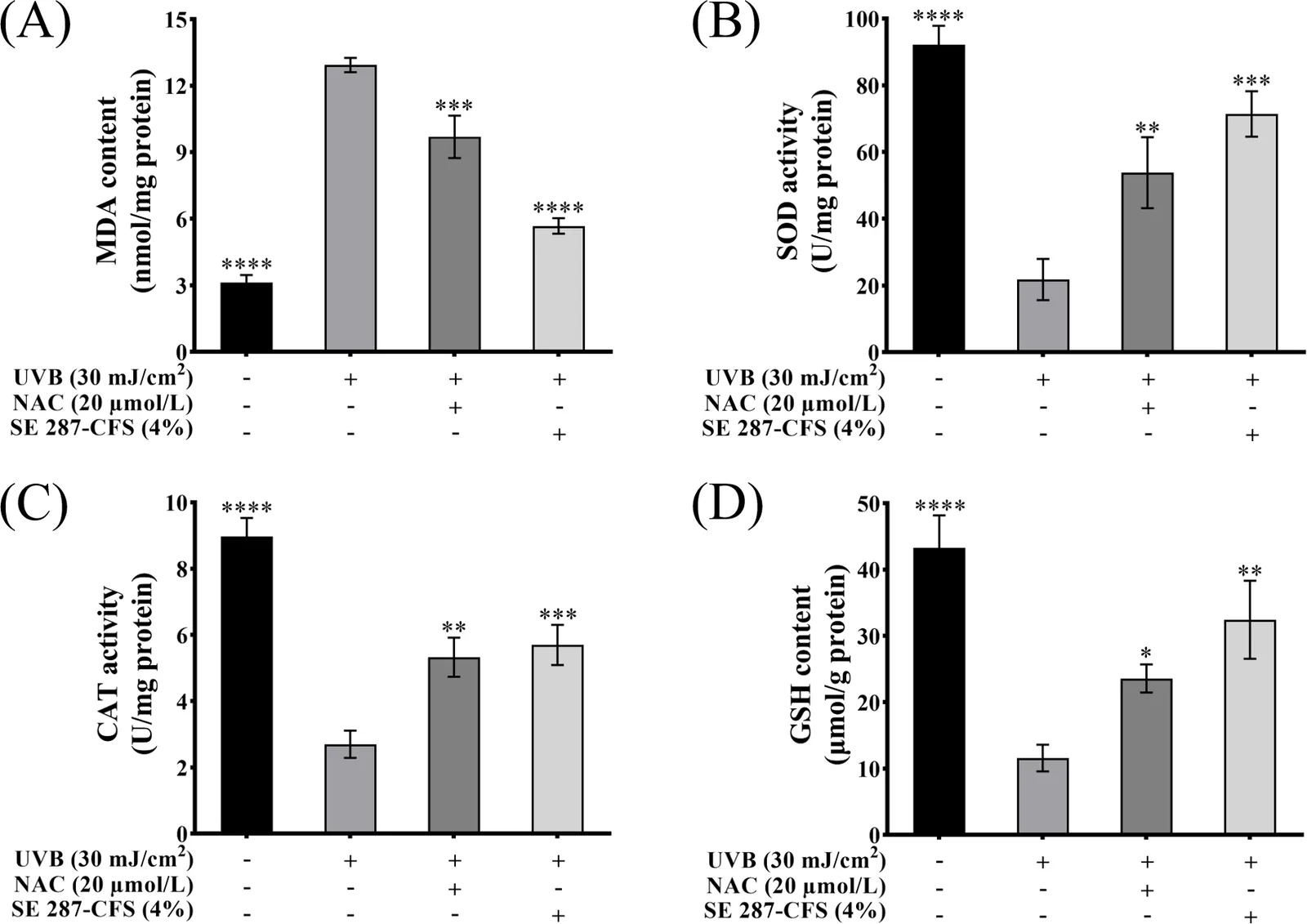
Changes of intracellular MDA and antioxidant defense system after UVB irradiation by SE 287-CFS. (**A**) MDA content of HaCaT cells in each group. (**B**) SOD activity of HaCaT cells in each group. (**C**) CAT activity of HaCaT cells in each group. (**D**) GSH content of HaCaT cells in each group. Data are expressed as mean ± SD from three biological replicates. *P < 0.05, **P < 0.01, ***P < 0.001, ****P < 0.0001 vs 30 mJ/cm^2^ UVB group as determined by one-way ANOVA.

To evaluate the antioxidative properties of SE 287-CFS against UVB-induced oxidative stress in HaCaT cells, MDA, SOD, CAT, and GSH were used as indicators, with results shown in Fig. 3A through D. Compared to the control group (cells without UVB exposure or any treatment), UVB stimulation significantly increased MDA content and decreased SOD activity, CAT activity, and GSH content (*P* < 0.0001). After UVB irradiation, MDA content increased to (12.93 ± 0.32) nmol/mg protein, while SOD and CAT activities decreased to (21.79 ± 6.18) U/mg protein and (2.70 ± 0.41) U/mg protein, respectively, and GSH content decreased to (11.58 ± 2.01) μmol/g protein. A 4% concentration of SE 287-CFS significantly reduced MDA content to (5.68 ± 0.35) nmol/mg protein (*P* < 0.0001), and increased SOD activity, CAT activity, and GSH content to (71.41 ± 6.78) U/mg protein (*P* = 0.0002), (5.70 ± 0.61) U/mg protein (*P* = 0.0007), and (32.41 ± 5.89) μmol/g protein (*P* = 0.0011), respectively.

### Effect of SE 287-CFS on NO production and the secretion of cytokines

**Fig 4:**
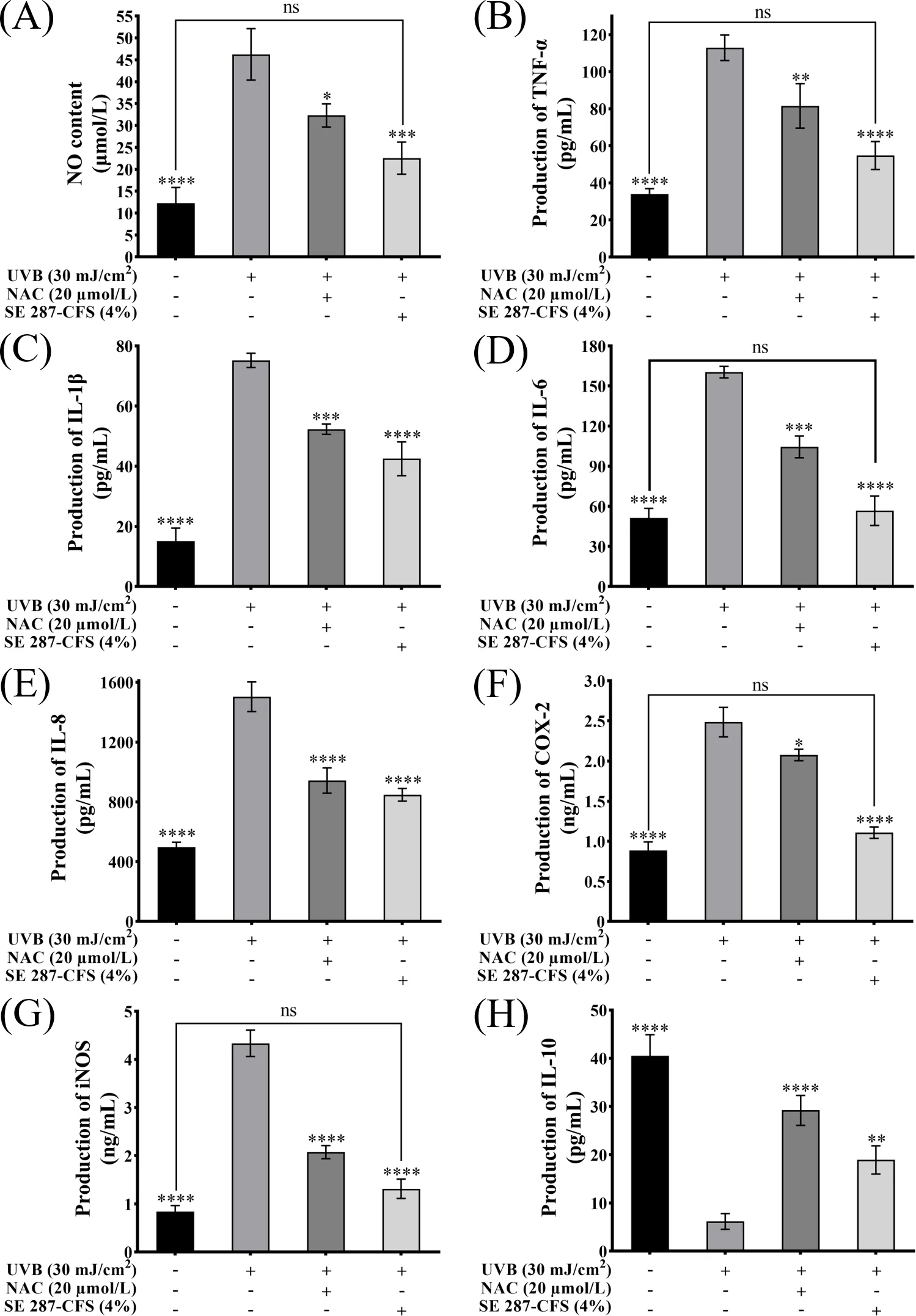
The effect of SE 287-CFS diminished UVB-induced pro-inflammatory response by modulating cytokines and mediators of inflammation. (**A**) NO content in HaCaT cells of different treatment groups. ELISA assay of (**B**) TNF-α, (**C**) IL-1β, (**D**) IL-6, (**E**) IL-8, (**F**) COX-2, (**G**) iNOS, and (**H**) IL-10 in HaCaT cells of different treatment groups. Data are expressed as mean ± SD from three biological replicates. *P < 0.05, **P < 0.01, ***P < 0.001, ****P < 0.0001 vs 30 mJ/cm^2^ UVB group as determined by one-way ANOVA. ns indicated no significant difference.

NO is commonly considered an important indicator for assessing the onset and treatment of chronic inflammation. Cytokines are key mediators of cell interactions and play essential roles in immune regulation, inflammatory responses, apoptosis, and cell growth and survival. Most cytokines investigated for their anti-inflammatory effects include pro-inflammatory cytokines, such as TNF-α, IL-1β, IL-6, IL-8, COX-2, and iNOS. This study evaluated the effects of SE 287-CFS on the content and expression of various cytokines in a UVB-induced inflammation model in HaCaT cells. As shown in Fig. 4, intracellular NO content and cytokine expression levels were measured by ELISA. Under 30 mJ/cm^2^ UVB irradiation, expression levels of NO, TNF-α, IL-1β, IL-6, IL-8, COX-2, and iNOS significantly increased (*P* < 0.0001), while IL-10 expression levels significantly decreased (*P* < 0.0001). Treatment with 4% SE 287-CFS significantly reduced UVB-induced upregulation of NO, TNF-α, IL-1β, IL-6, IL-8, COX-2, and iNOS expression levels in HaCaT cells (Fig. 4A through G). Compared with the UVB group, 4% SE 287-CFS significantly reduced NO production from 46.23 ± 5.86 to 22.55 ± 3.67 µmol/L (*P* = 0.0005). It also significantly decreased the expression of TNF-α, IL-1β, IL-6, IL-8, COX-2, and iNOS (*P* < 0.0001). Furthermore, treatment with 4% SE 287-CFS reduced the expression levels of NO, TNF-α, IL-6, COX-2, and iNOS to levels not significantly different from those in the control group. The expression level of the anti-inflammatory cytokine IL-10 was also measured, with results shown in Fig. 4H. Treatment with 4% SE 287-CFS significantly upregulated the UVB-induced downregulation of IL-10, elevating its expression from (6.17 ± 1.63) to (18.93 ± 2.92) pg/mL (*P* = 0.005). These results suggest that SE 287-CFS may mitigate UVB-induced inflammatory responses.

### Effect of SE 287-CFS on the inhibition of MAPK signaling activation

**Fig 5:**
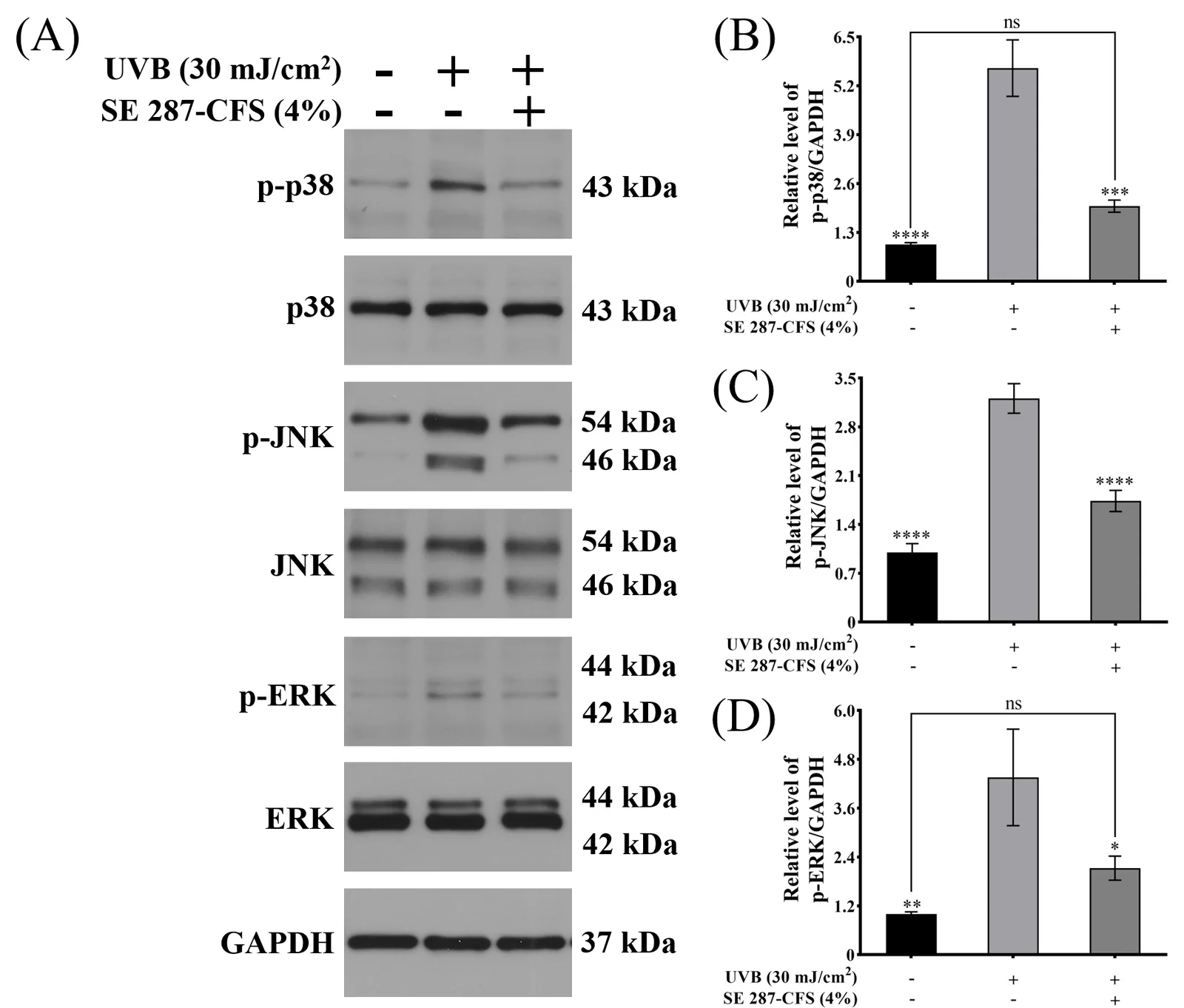
Effect of SE 287-CFS on the activation of MAPK signaling pathway in UVB-irradiated HaCaT cells. (**A**) Western blotting analysis of the production of MAPK and phosphorylated MAPK. ImageJ-normalized quantitative analysis of the p-p38/GAPDH (**B**), p-JNK/GAPDH (**C**), and p-ERK/GAPDH (**D**) ratios from immunoblots in panel **A**. Data are expressed as mean ± SD from three biological replicates. *P < 0.05, **P < 0.01, ***P < 0.001, ****P < 0.0001 vs 30 mJ/cm^2^ UVB group as determined by one-way ANOVA. ns indicated no significant difference.

Members of the MAPK family, including p38, JNK, and ERK, are key initiators of inflammation-inducing signals and serve as therapeutic targets for inflammatory diseases. We investigated the effect of 4% SE 287-CFS on the phosphorylation levels of MAPK family members p38, JNK, and ERK in HaCaT cells induced by 30 mJ/cm^2^ UVB irradiation. As shown in Fig. 5, western blotting analysis demonstrated that UVB irradiation significantly activated the phosphorylation of JNK, p38, and ERK in the MAPK signaling pathway. Furthermore, treatment with SE 287-CFS significantly inhibited the phosphorylation of p38 (*P* = 0.0001), JNK (*P* < 0.0001), and ERK (*P* = 0.0194) in UVB-induced cells.

### Effect of SE 287-CFS on the inhibition of NF-κB signaling activation

**Fig 6:**
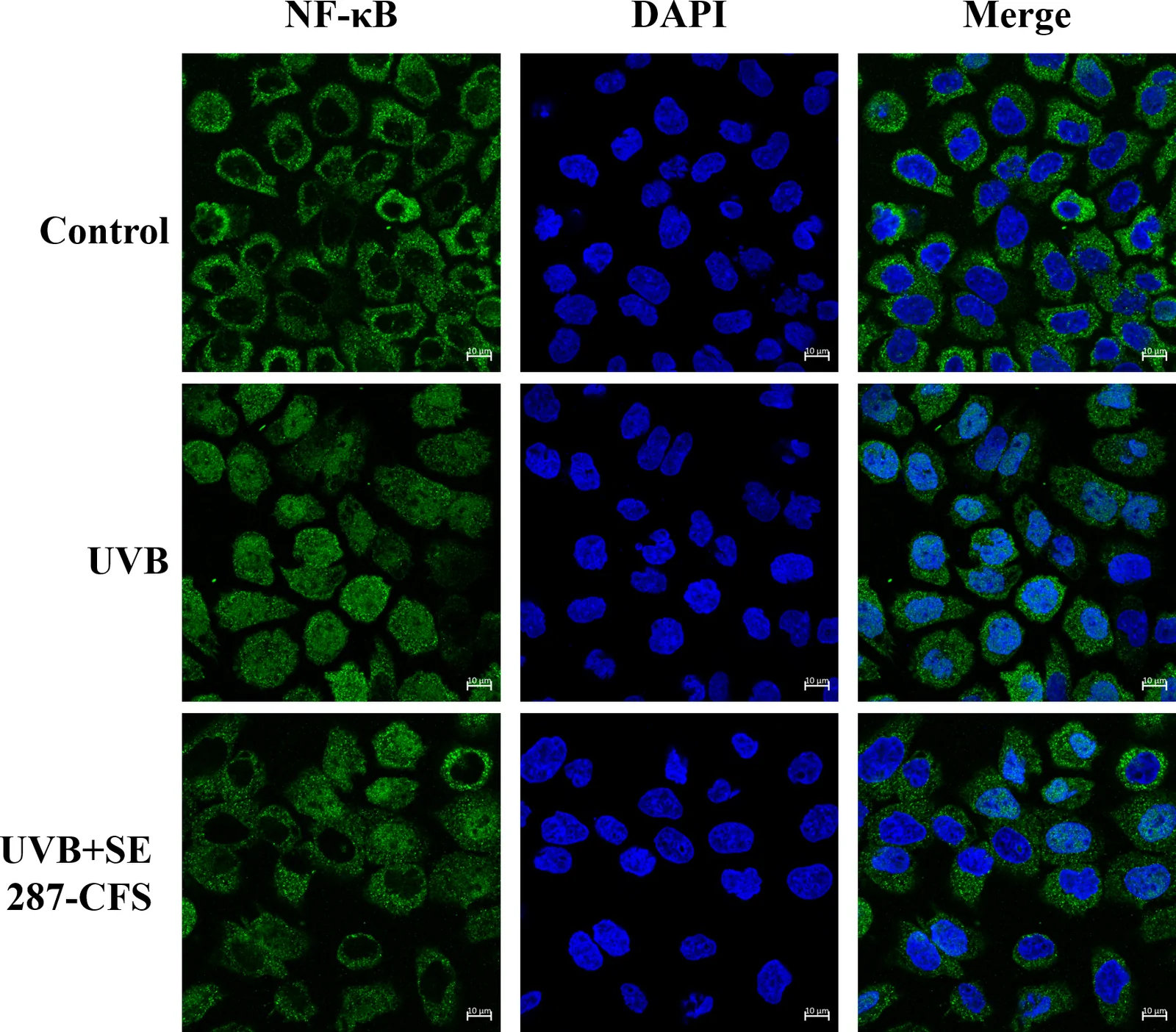
Effect of SE 287-CFS on NF-κB nuclear translocation in UVB-induced HaCaT cells. The nuclear localization of NF-κB was determined using fluorescence microscopy after staining with DAPI (nucleus, blue), anti-p65, and CoraLite488-conjugated Goat Anti-Rabbit IgG (green) secondary antibody, bars = 10 µm.

As shown in Fig. 6, NF-κB fluorescence was weak in the nucleus of the control group, but UVB treatment markedly increased the fluorescence signal in the nuclear regions. The accumulated NF-κB in the nucleus could significantly promote the expression of inflammatory cytokines. However, in the SE 287-CFS-treated group, a reduction in NF-κB fluorescence was observed in the nuclear region, indicating that SE 287-CFS could mitigate NF-κB translocation from the cytoplasm to the nucleus. This suggests a role in slowing inflammation in UVB-induced HaCaT cells, consistent with the western blot results.

**Fig 7:**
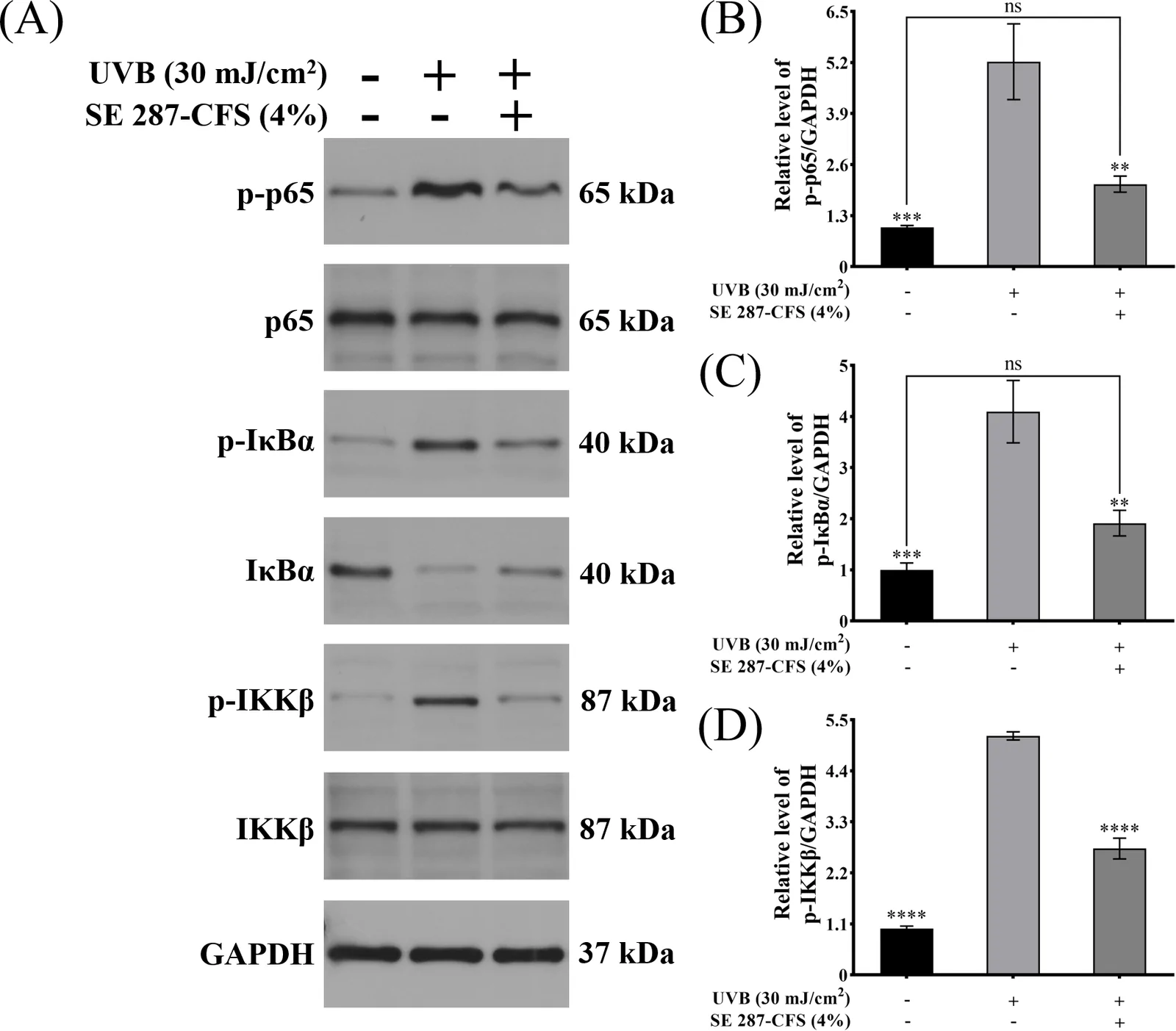
Effect of SE 287-CFS on the NF-κB signaling pathway in UVB-irradiated HaCaT cells. (**A**) Western blotting analysis on production of NF-κB and phosphorylated NF-κB. ImageJ normalized quantitative analysis of the p-p65/GAPDH (**B**), p-IκBα/GAPDH (**C**), and p-IKKβ/GAPDH (**D**) ratios from immunoblots in panel **A**. Data are expressed as mean ± SD from three biological replicates. **P < 0.01, ***P < 0.001, ****P < 0.0001 vs 30 mJ/cm^2^ UVB group as determined by one-way ANOVA. ns indicated no significant difference.

Previous studies have shown that NF-κB plays a critical role in regulating inflammation triggered by external stimuli. Therefore, it is essential to investigate how SE 287-CFS regulates NF-κB activation. We evaluated the effect of 4% SE 287-CFS on the phosphorylation of key proteins (p65, IκBα, and IKKβ) in the NF-κB pathway in HaCaT cells exposed to 30 mJ/cm^2^ UVB irradiation, as shown in Fig. 7. After UVB irradiation, phosphorylation levels of p-p65 (*P* = 0.0002), p-IκBα (*P* = 0.0002), and p-IKKβ (*P* < 0.0001) significantly increased, indicating NF-κB dissociation and activation. However, in UVB-induced cells, SE 287-CFS treatment significantly reduced the phosphorylation of p65 (*P* = 0.0013), IκBα (*P* = 0.0011), and IKKβ (*P* < 0.0001) compared to the UVB-only group.

### SE 287-CFS reinforced epidermal barrier function by suppressing pro-inflammatory genes and promoting epidermal cell renewal

**Fig 8:**
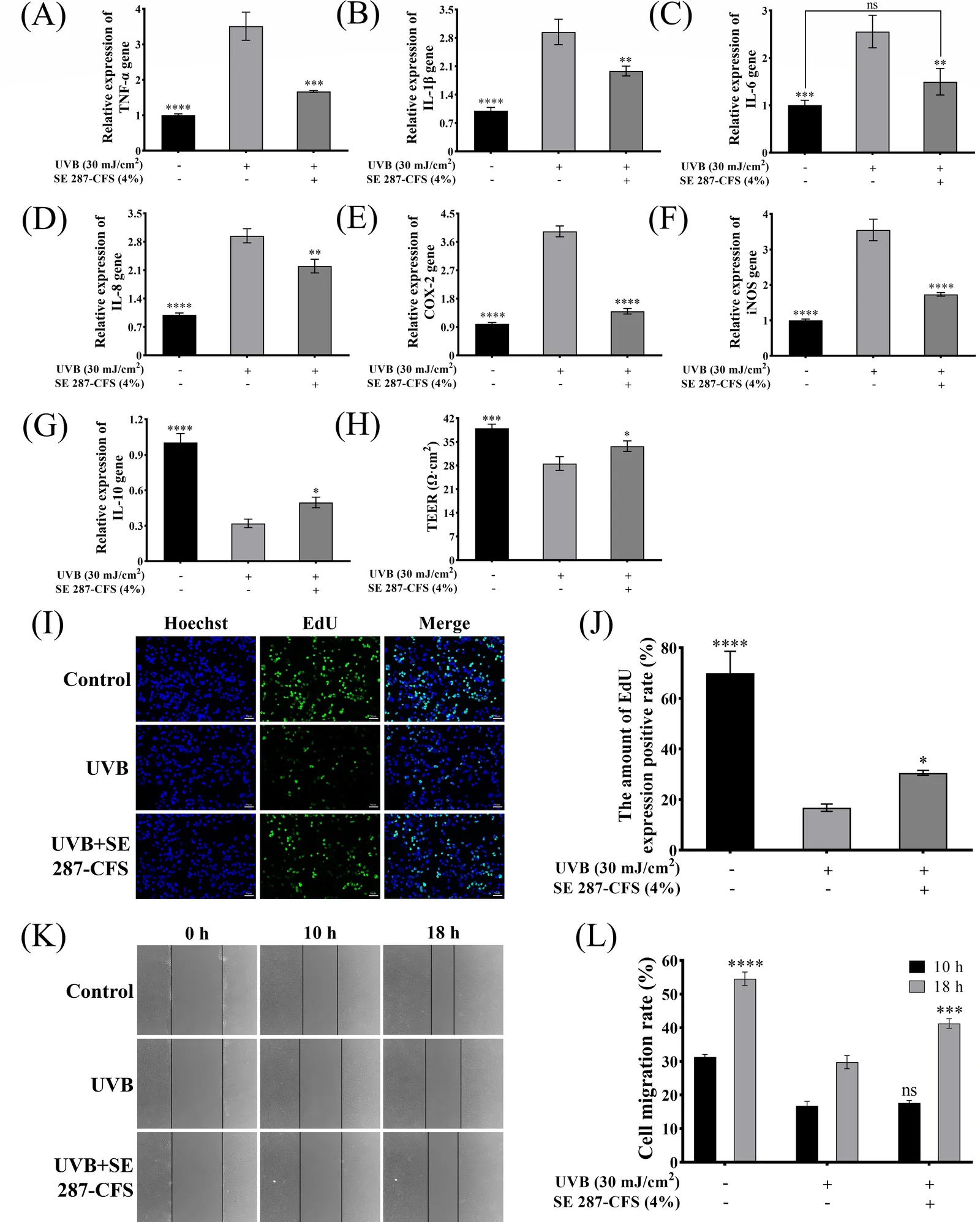
SE 287-CFS enhanced skin barrier function. (**A–G**) The mRNA levels of TNF-α, IL-1β, IL-6, IL-8, COX-2, iNOS, and IL-10 in HaCaT cells treated with cells without any treatment (control), 30 mJ/cm^2^ UVB, 30 mJ/cm^2^ UVB plus 4% concentration of SE 287-CFS for 18 h were analyzed by qPCR in comparison with the mRNA level of the housekeeping gene GAPDH. (**H**) TEER value of HaCaT cell monolayers following 18 h treatment with 4% concentration of SE 287-CFS. (**I**) and (**J**) The EdU incorporation of HaCaT cells treated with control, 30 mJ/cm^2^ UVB, 30 mJ/cm^2^ UVB plus 4% concentration of SE 287-CFS for 18 h, bars = 50 µm. (**K**) Representative images and HaCaT cells scratch assay results. (**L**) Cell migration rate of HaCaT cells in different groups at 10 and 18 h. Data are expressed as mean ± SD from three biological replicates. *P < 0.05, **P < 0.01, ***P < 0.001, ****P < 0.0001 vs 30 mJ/cm^2^ UVB group as determined by one-way ANOVA. ns indicated no significant difference.

UVB irradiation causes both an inflammatory response in the epidermis and barrier dysfunction. To investigate the effects of SE 287-CFS on epidermal barrier function, we examined the interaction between HaCaT cells and SE 287-CFS. As shown in Fig. 8A through F, SE 287-CFS significantly reduced the expression of pro-inflammatory genes (TNF-α, IL-1β, IL-6, IL-8, COX-2, and iNOS) in UVB-treated HaCaT cells. Additionally, SE 287-CFS significantly increased the expression of the anti-inflammatory gene IL-10 in UVB-treated HaCaT cells (Fig. 8G). Next, we investigated the role of SE 287-CFS in tight junction formation and permeability in HaCaT monolayers by measuring TEER across these layers. As shown in Fig. 8H, SE 287-CFS significantly enhanced TEER in UVB-irradiated HaCaT cells (*P* = 0.0203). Furthermore, we examined whether SE 287-CFS could promote epidermal cell renewal. As shown in Fig. 8I through L, SE 287-CFS significantly increased the proliferation and migration of UVB-induced HaCaT cells. These results suggest that SE 287-CFS promotes epidermal cell renewal.

### Non-targeted metabolomics analysis of SE 287-CFS

**Fig 9:**
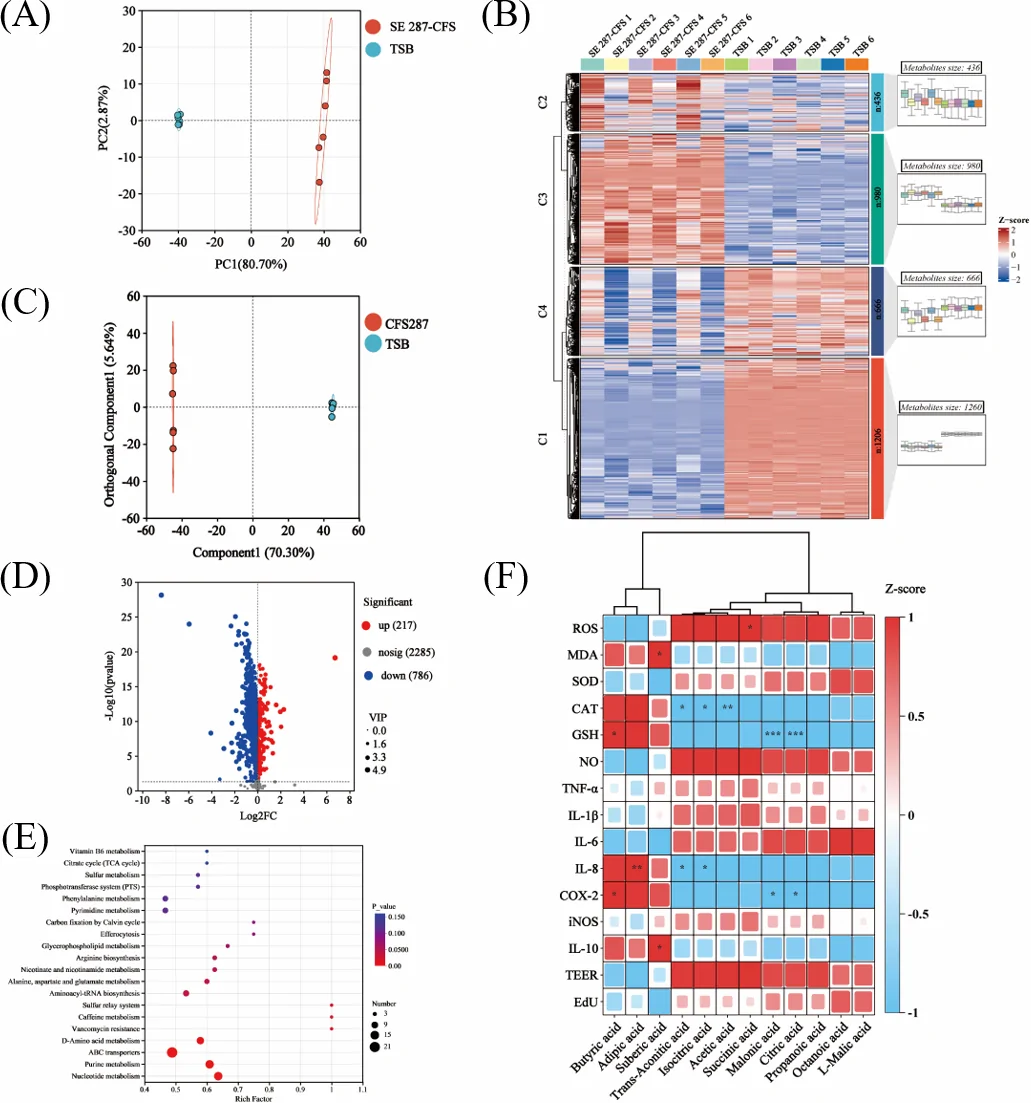
Metabolomics analysis of SE 287-CFS. (**A**) PCA analysis of metabolites from SE 287-CFS and TSB. (**B**) Trend heatmap of metabolites from SE 287-CFS and TSB. (**C**) OPLS-DA analysis of differential metabolites between SE 287-CFS and TSB. (**D**) Volcano plot of differential metabolites between SE 287-CFS and TSB. (**E**) Bubble plot of KEGG metabolic pathway effects in SE 287-CFS and TSB groups. (**F**) Correlation analysis of organic acids with oxidative stress, inflammation, and skin barrier indicators in SE 287-CFS, *P < 0.05, **P < 0.01, ***P < 0.001.

The metabolites of SE 287-CFS and TSB were analyzed by LC-MS. The PCA plot of metabolites from SE 287-CFS and TSB was shown in Fig. 9A, demonstrating a clear separation between the two groups. The heatmap further showed significant metabolic differences between the SE 287-CFS and TSB groups (Fig. 9B). OPLS-DA modeling was used to further investigate the differential metabolites. To ensure result reliability, a permutation test with 200 iterations was conducted on the OPLS-DA model. The OPLS-DA analysis of differential metabolites between SE 287-CFS and TSB in Fig. 9C, showing complete separation between the two groups. Differential metabolites were selected based on the variable importance projection (VIP) principle, with VIP > 1, *P* value < 0.05, and fold change (FC) > 1.0. A total of 1,003 differential metabolites were identified by comparison with databases. Compared to TSB, SE 287-CFS upregulated 217 metabolites and downregulated 786, primarily organic acids, peptides, and carbohydrates (Fig. 9D). Figure 9E displays the top 20 KEGG pathways annotated for the differential metabolites. The top five pathways with the highest concentrations of differential metabolites are nucleotide metabolism, purine metabolism, ABC transporters, D-amino acid metabolism, and aminoacyl-tRNA biosynthesis. The correlation analysis of organic acids in SE 287-CFS metabolites with oxidative stress, inflammation, and skin barrier markers is shown in Fig. 9F. Butyric acid and adipic acid were negatively correlated with ROS, NO, TNF-α, IL-1β, IL-6, and iNOS, and positively correlated with CAT, GSH, and IL-10, suggesting their roles in antioxidation and anti-inflammation. Furthermore, succinic acid, acetic acid, propanoic acid, malonic acid, trans-aconitic acid, isocitric acid, citric acid, octanoic acid, and L-malic acid were negatively correlated with MDA, IL-8, and COX-2, and positively correlated with SOD, TEER, and EdU, indicating that these organic acids contribute primarily to skin barrier repair.

## DISCUSSION

Skin is a superficial organ in direct contact with the surrounding environment, serving as the first line of defense against external hazards. However, it is also vulnerable to external stressors. UV radiation is a harmful environmental stressor that causes significant damage to the skin (31). Repeated and chronic UV exposure induces various skin damages, including photoaging, inflammation, oxidative stress, immune suppression, and skin tumor formation (32–35). Several studies have shown that *S. epidermidis* protects skin health by promoting immunity, repair, antimicrobial defense, and anti-tumor activity (36). However, the beneficial effects of commensal *S. epidermidis* on UVB-induced skin damage remain unclear. In this study, we established a UVB-induced HaCaT cell model, and the protective effect and mechanism of SE 287-CFS were explored. Our results indicated that SE 287-CFS significantly inhibited oxidative stress, reduced inflammation, and repaired the skin barrier, highlighting its potential in protecting the skin from UVB-induced photodamage.

UVB exposure induces the production of ROS, which include unstable oxygen radicals, such as superoxide anions (O2·^−^), hydrogen peroxide (H_2_O_2_), and hydroxyl radicals (OH·). ROS homeostasis is crucial for cell survival and protection against cellular damage. Excessive ROS damages cellular proteins, lipids, and DNA while inhibiting the antioxidant defense system, leading to oxidative stress and apoptosis (37). In our experiment, UVB irradiation significantly increased ROS levels in HaCaT cells, corresponding to a significant decrease in cell viability and an increase in the expression of pro-inflammatory genes, as shown in Fig. 1A through C. These findings are consistent with previous studies highlighting the detrimental effects of UVB-induced oxidative stress on skin cells (38). SE 287-CFS effectively mitigated ROS accumulation, as evidenced by the reduction in ROS fluorescence intensity (Fig. 2). Therefore, SE 287-CFS inhibits the harmful effects of oxidative stress by reducing ROS production. The ability of *S. epidermidis* to reduce ROS accumulation has been reported in a previous study (39).

UVB irradiation not only elevates ROS levels but also induces lipid peroxidation, commonly assessed by measuring MDA levels, which reflects the extent of cellular damage (40). As shown in Fig. 3, UVB irradiation significantly increased MDA content in HaCaT cells, indicating exacerbated oxidative stress, while SE 287-CFS significantly reduced MDA levels. Oxidative stress develops when an imbalance occurs between antioxidants and ROS. Further investigation revealed that in response to ROS, the body’s natural antioxidant system, primarily composed of SOD, CAT, and GSH, scavenges O2·^−^, H_2_O_2_, and OH· through catalytic reactions to maintain homeostasis (41, 42). Antioxidant enzymes that scavenge reactive oxygen in the skin are depleted by prolonged UV exposure. However, SE 287-CFS can restore antioxidant enzyme levels. These results suggest that SE 287-CFS has a positive effect on oxidative stress and helps restore cellular redox balance. SE 287-CFS plays a protective role by inhibiting the ROS signaling pathway.

UVB irradiation stimulates the production of NO, which is associated with various inflammatory skin diseases, and increases the expression of inflammatory cytokines, such as IL-1β and TNF-α, key players in the pathogenesis of UVB-induced skin inflammation (43). NO is produced by iNOS, which is strongly induced by inflammatory cytokines in cells. Inhibiting these cytokines can improve skin damage (44). As a signaling molecule, NO has cytotoxic effects on cells and contributes to the inflammatory response in tissue injury (45). Furthermore, UV-induced acute skin inflammation triggers the release of these cytokines, which activate MAPK and NF-κB signaling pathways (46). In our study, SE 287-CFS significantly reduced the expression of pro-inflammatory cytokines and increased the expression of the anti-inflammatory cytokine IL-10. This modulation of cytokine expression highlights the probiotic properties of *S. epidermidis* CCSM0287 in balancing the inflammatory response, a mechanism previously documented in skin-associated bacteria (23, 47).

ROS induced by UVB exposure activate cytoplasmic signaling pathways, including MAPK, JAK-STAT, PI3K-AKT, NF-κB, and Nrf-2, which are involved in cell survival, death, and inflammation (29, 48). These pathways may mediate oxidative stress-induced skin inflammation. The MAPK pathway regulates key cellular processes, including stress response, gene induction, and inflammation, and consists of a family of highly conserved serine/threonine protein kinases (49). In response to inflammatory signals, the MAPK cascade is activated through phosphorylation of p38, ERK, and JNK, which are involved in cell proliferation, apoptosis, and inflammation. UV-induced ROS upregulation activates MAPK phosphorylation, leading to IκB degradation through both IKK-dependent and independent pathways, which activates NF-κB and promotes cytokine secretion (50). Recent studies have shown that ROS is involved in both MAPK and NF-κB activation, with MAPK signaling playing a key role in NF-κB activation (51). Therefore, inhibiting the MAPK and NF-κB pathways protects against inflammation. p65, a member of the NF-κB family, is located in the cytoplasm. However, when stimulated by cytokines or exogenous inducers, receptor configuration changes activate IκBα kinase, which phosphorylates IκBα. After ubiquitination, phosphorylated IκBα is recognized and degraded by the 26S proteasome. NF-κB is then released from the NF-κB/IκBα complex in the cytoplasm, enters the nucleus, binds to DNA, and regulates the expression of inflammatory mediators (52). Phosphorylation of p65 is essential for its nuclear translocation in the NF-κB signaling cascade. Our results showed that SE 287-CFS significantly inhibited the phosphorylation of JNK, p38, and ERK in UVB-induced HaCaT cells. Additionally, we observed that SE 287-CFS inhibited UVB-induced phosphorylation of p65, IκBα, and IKKβ, as well as the nuclear translocation of NF-κB in HaCaT cells. These findings demonstrated that SE 287-CFS inhibited the inflammatory cascade following UVB irradiation, preventing further skin damage.

The skin barrier not only prevents physical damage but also blocks microbial invasion by excluding bacteria, viruses, fungi, and other harmful substances (53). It maintains the skin’s microecological balance, thereby preventing infections and inflammation associated with barrier disruption (54). UVB exposure impairs skin barrier function and, if left untreated, may lead to the development of skin diseases (55). TEER is a key indicator of cellular barrier integrity, reflecting both the tightness of intercellular junctions and cellular permeability. Higher TEER values indicated stronger barrier function. In our study, SE 287-CFS significantly restored the reduced TEER values in UVB-treated HaCaT cells. Moreover, epidermal barrier damage and cutaneous inflammation are mutually reinforcing (56, 57). This study demonstrated that SE 287-CFS suppressed inflammation and promoted epidermal cell renewal. These dual effects of SE 287-CFS may accelerate the repair of epidermal barrier damage. These findings align with the established role of *S. epidermidis* in maintaining the skin barrier. Previous studies have shown that symbiotic *S. epidermidis* contributes to barrier integrity, promotes epithelial regeneration after injury, and accelerates wound healing (36, 58).

Non-targeted metabolomics analysis identified organic acids as the primary differential metabolites between SE 287-CFS and TSB, most of which were short-chain fatty acids (SCFAs). Correlation analysis of organic acids with markers of oxidative stress, inflammation, and skin barrier function indicated that butyric acid and adipic acid exerted antioxidative and anti-inflammatory effects, and other organic acids contributed to skin barrier repair. Short-chain fatty acids are important metabolites not only for gut microbiota but also for skin microbiota to exert their functions (59). Studies have shown that SCFAs exhibit antibacterial activity, inhibit biofilm formation, lower skin pH to create an acidified barrier, and exert anti-inflammatory effects (23, 60). Butyrate produced by *S. epidermidis* ATCC 12228 attenuated UVB-induced IL-6 expression in mouse skin, thereby suppressing inflammatory responses (18). Clinically isolated *S. epidermidis* strains primarily produce butyrate, highlighting the widespread relevance of this metabolite (61). The oligopeptides in the fermentation supernatant of *S. epidermidis* ATCC 12228 significantly reduced the expression of pro-inflammatory cytokines IL-1β and IL-6 (62). *S. epidermidis* CCSM0287 mainly produced acetic acid, isovaleric acid, and butyric acid in TSB-based medium (63). The fermentation supernatant of *S. epidermidis* CCSM0287 also had the antioxidant enzymes, including SOD and GSH-PX activities, 33.51 ± 0.44 U/mL and 17.77 ± 2.64 U/mL, respectively. These bioactive components, such as SCFAs, other organic acids, oligopeptides, and the antioxidant enzymes, and more than that, may act synergistically to alleviate oxidative stress, suppress pro-inflammatory cytokines, and promote barrier repair.

**Fig 10:**
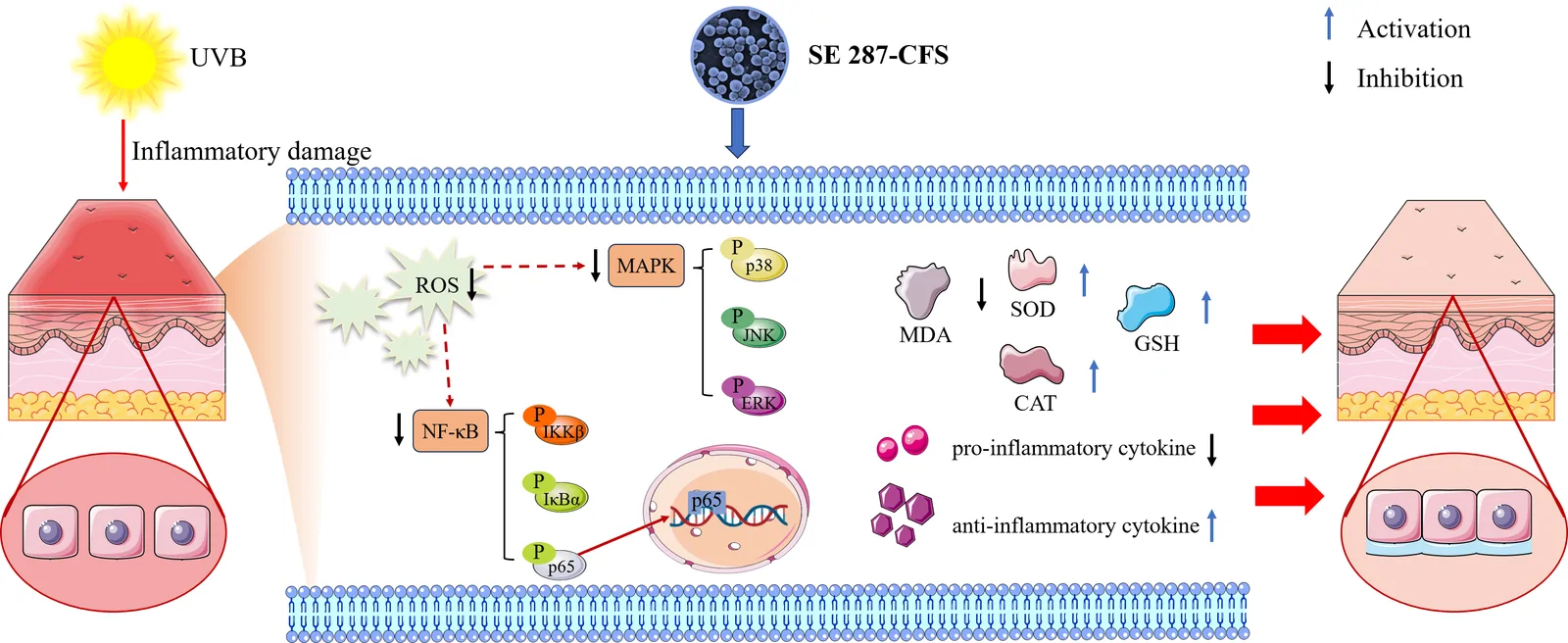
Schematic diagram illustrating the mechanism by which SE 287-CFS mitigated UVB-induced photodamage. In UVB-induced HaCaT cells, SE 287-CFS reduced oxidative stress and inflammation through the ROS/MAPK/NF-κB signaling pathway and enhanced skin barrier function.

In conclusion, our study demonstrated that SE 287-CFS protected HaCaT cells from UVB-induced damage by alleviating oxidative stress and inflammation through modulation of the ROS/MAPK/NF-κB signaling pathway. SE 287-CFS also promoted keratinocyte proliferation, migration, and TEER recovery, suggesting a beneficial role in restoring skin barrier function (Fig. 10). Our results expanded current understanding of the photoprotective potential of postbiotics derived from skin commensals and provided a basis for the future screening of microbiome-derived bioactive agents for skin protection. However, this study was limited to a 2D HaCaT cell model, and the candidate metabolites identified by metabolomic analysis still require causal validation. Future studies should focus on validating these effects in 3D skin models and *in vivo* systems, as well as clarifying the causal contributions of candidate metabolites to the observed photoprotective effects.

## Data Availability

The complete genome sequence of *S. epidermidis* CCSM0287 is available in GenBank under accession numbers CP110611 to CP110614, BioProject accession number PRJNA893857, BioSample accession number SAMN31433203, and Sequence Read Archive (SRA) accession number SRP422170. *S. epidermidis* CCSM0287 will be made available to researchers in this field for non-commercial research upon email request to the corresponding author of this study (Dr. Shao, teamo-lily@163.com). Transfer of the strain may be subject to a reasonable material transfer agreement and/or non-disclosure agreement, as well as applicable institutional, biosafety, and shipping regulations.

## References

[1] Harris-Tryon TA, Grice EA. 2022. Microbiota and maintenance of skin barrier function. Science 376:940–945. doi:10.1126/science.abo0693 35617415

[2] Grice EA, Segre JA. 2011. The skin microbiome. Nat Rev Microbiol 9:244–253. doi:10.1038/nrmicro2537 PMC3535073 21407241

[3] Gribonika I, Band VI, Chi L, Perez-Chaparro PJ, Link VM, Ansaldo E, Oguz C, Bousbaine D, Fischbach MA, Belkaid Y. 2025. Skin autonomous antibody production regulates host–microbiota interactions. Nature 638:1043–1053. doi:10.1038/s41586-024-08376-y PMC11864984 39662506

[4] Ochlich D, Rademacher F, Drerup KA, Gläser R, Harder J. 2023. The influence of the commensal skin bacterium *Staphylococcus epidermidis* on the epidermal barrier and inflammation: implications for atopic dermatitis. Exp Dermatol 32:555–561. doi:10.1111/exd.14727 36484602

[5] Zhou W, Spoto M, Hardy R, Guan C-H, Fleming E, Larson PJ, Brown JS, Oh J. 2020. Host-specific evolutionary and transmission dynamics shape the functional diversification of *Staphylococcus epidermidis* in human skin. Cell 180:454–470. doi:10.1016/j.cell.2020.01.006 PMC7192218 32004459

[6] Li D-Q, Wang W, Wu Y-L, Ma X-J, Zhou W-B, Lai Y-P. 2019. Lipopeptide 78 from *Staphylococcus epidermidis* activates β-catenin to inhibit skin inflammation. The J Immunol 202:1219–1228. doi:10.4049/jimmunol.1800813 30642981

[7] Nakatsuji T, Chen TH, Butcher AM, Trzoss LL, Nam SJ, Shirakawa KT, Zhou W, Oh J, Otto M, Fenical W, Gallo RL. 2018. A commensal strain of *Staphylococcus epidermidis* protects against skin neoplasia. Sci Adv 4:eaao4502. doi:10.1126/sciadv.aao4502 PMC5834004 29507878

[8] Linehan JL, Harrison OJ, Han SJ, Byrd AL, Vujkovic-Cvijin I, Villarino AV, Sen SK, Shaik J, Smelkinson M, Tamoutounour S, Collins N, Bouladoux N, Dzutsev A, Rosshart SP, Arbuckle JH, Wang CR, Kristie TM, Rehermann B, Trinchieri G, Brenchley JM, O’Shea JJ, Belkaid Y. 2018. Non-classical immunity controls microbiota impact on skin immunity and tissue repair. Cell 172:784–796. doi:10.1016/j.cell.2017.12.033 PMC6034182 29358051

[9] Brown MM, Horswill AR. 2020. *Staphylococcus epidermidis*-skin friend or foe? PLoS Pathog 16:e1009026. doi:10.1371/journal.ppat.1009026 PMC7660545 33180890

[10] Choi SI, Han HS, Kim JM, Park G, Jang YP, Shin YK, Ahn HS, Lee SH, Lee KT. 2021. *Eisenia bicyclis* Extract repairs UVB-induced skin photoaging *in vitro* and *in vivo*: photoprotective effects. Mar Drugs 19:693. doi:10.3390/md19120693 PMC8709268 34940692

[11] Dou S, Pan W, Pan X, Li Y, Mao J, Cheng J, Yan X, Wang T, Zhou X, Zhang SY, Zhang G. 2025. The organic acid metabolites of *Bifidobacterium longum* subsp. iuvenis alleviate ultraviolet irradiation-induced photoaging. Microbiol Res 294:128100. doi:10.1016/j.micres.2025.128100 39955985

[12] Melo CPB, Saito P, Vale DL, Rodrigues CCA, Pinto IC, Martinez RM, Bezerra JR, Baracat MM, Verri WA Jr, Fonseca-Bazzo YM, Georgetti SR, Casagrande R. 2021. Protective effect of oral treatment with *Cordia verbenacea* extract against UVB irradiation deleterious effects in the skin of hairless mouse. J Photoch Photobio B 216:112151. doi:10.1016/j.jphotobiol.2021.112151 33581679

[13] Cheon SJ, Kim JY, Kim GW, Lee JY, Kim SH. 2023. Lactic acid bacteria and their lysate ameliorate skin inflammation in a UVB irradiation-induced skin photoaging mouse model. Physiology 38:5766749. doi:10.1152/physiol.2023.38.S1.5766749

[14] Ácsová A, Hojerová J, Martiniaková S. 2022. Efficacy of postbiotics against free radicals and UV radiation. Chem Pap 76:2357–2364. doi:10.1007/s11696-021-02018-7

[15] Tyara Simbara A, Faridatul Habibah F, Hertadi R. 2025. Rhamnolipid-modified PHB–ectoine nanoparticles for multifunctional skin protection against UVB, irritation, and bacteria. ACS Omega 10:12200–12213. doi:10.1021/acsomega.4c10583 PMC11966311 40191376

[16] Zhang L-X, Gu W-J, Liu T, Pei H-N, Ma Y-L, Zhao Y, Huang S, Chen M-L. 2025. NDRG2 deficiency exacerbates UVB-induced skin inflammation and oxidative stress damage. Inflammation 48:1313–1325. doi:10.1007/s10753-024-02121-3 39145786

[17] Song S-J, Li F-X, Zhao B-X, Zhou M, Wang X-B. 2025. Ultraviolet light causes skin cell senescence: from mechanism to prevention principle. Advanced Biology 9:2400090. doi:10.1002/adbi.202400090 39364703

[18] Sun Q-R, Fang J-X, Wang Z-W, Song Z-X, Geng J-M, Wang D-D, Wang C-T, Li M. 2022. Two *Laminaria japonica* fermentation broths alleviate oxidative stress and inflammatory response caused by UVB damage: photoprotective and reparative effects. Mar Drugs 20:650. doi:10.3390/md20100650 PMC9605345 36286472

[19] Smith ML, O’Neill CA, Dickinson MR, Chavan B, McBain AJ. 2023. Exploring associations between skin, the dermal microbiome, and ultraviolet radiation: advancing possibilities for next-generation sunscreens. Front Microbiomes 2:1102315. doi:10.3389/frmbi.2023.1102315 PMC12993599 41853364

[20] Stacy A, Belkaid Y. 2019. Microbial guardians of skin health. Science 363:227–228. doi:10.1126/science.aat4326 30655428

[21] Patra V, Wagner K, Arulampalam V, Wolf P. 2019. Skin Microbiome modulates the effect of ultraviolet radiation on cellular response and immune function. iScience 15:211–222. doi:10.1016/j.isci.2019.04.026 PMC6515114 31079025

[22] Wang Z-P, Choi JE, Wu CC, Nardo AD. 2018. Skin commensal bacteria *Staphylococcus epidermidis* promote survival of melanocytes bearing UVB‐induced DNA damage, while bacteria *Propionibacterium acnes* inhibit survival of melanocytes by increasing apoptosis. Photodermatol Photo 34:405–414. doi:10.1111/phpp.12411 29974533

[23] Keshari S, Balasubramaniam A, Myagmardoloonjin B, Herr DR, Negari IP, Huang C-M. 2019. Butyric Acid from probiotic *Staphylococcus epidermidis* in the skin microbiome down-regulates the ultraviolet-induced pro-inflammatory IL-6 cytokine via short-chain fatty acid receptor. Int J Mol Sci 20:4477. doi:10.3390/ijms20184477 PMC6769796 31514281

[24] Balasubramaniam A, Tang J, Herr DR, Huang C-M. 2025. Electrogenic *Staphylococcus epidermidis* diminishes the ultraviolet B-induced labile irons in mouse skin. Curr Microbiol 82:160. doi:10.1007/s00284-025-04145-w 40014179

[25] Gulcin İ, Alwasel SH. 2023. DPPH radical scavenging assay. Processes 11:2248. doi:10.3390/pr11082248

[26] Zhao L, Wang Y-M, Li Y. 2019. Antioxidant activity of graphene quantum dots prepared in different electrolyte environments. Nanomaterials 9:1708. doi:10.3390/nano9121708 PMC6955962 31795321

[27] Wang K-X, Pramod SN, Lin H, Chen G-Z, Li Z-X. 2021. Process optimization for preparation of hyaluronidase inhibitory hydrolysates with anti-allergic potential from *Salmo salar* processing by-products. ACS Food Sci Technol 1:1262–1273. doi:10.1021/acsfoodscitech.1c00152

[28] Ma L-J, Yang S-Z, Jiang H, Yuan C-Y, Chen H-D, Li Y, Shao L. 2023. Complete genome sequence of *Staphylococcus epidermidis* CCSM0287, isolated from healthy facial skin. Microbiol Resour Announc 12:e00131–23. doi:10.1128/mra.00131-23 PMC10112243 36939347

[29] Li Y-Y, Ma Y-G, Yao Y-K, Ru G-H, Lan C, Li L-Y, Huang T. 2024. Protective effect of isoquercitrin on UVB‐induced injury in HaCaT cells and mice skin through anti‐inflammatory, antioxidant, and regulation of MAPK and JAK2‐STAT3 pathways. Photochem Photobiol 100:1507–1518. doi:10.1111/php.13919 38337181

[30] Zhang M-Z, Qin H-G, Xiang L-J, An L-J, Zhang X-L, Li K-X, Wu K, Fei X-Y, Fan W-H, Xu X-Y, Xu P-F, Wu Y, Mu D. 2024. *Camellia sinensis* polysaccharide attenuates inflammatory responses via the ROS-mediated pathway by endocytosis. Int J Biol Macromol 267:131674. doi:10.1016/j.ijbiomac.2024.131674 38641285

[31] Elhabak M, Ibrahim S, Abouelatta SM. 2021. Topical delivery of l-ascorbic acid spanlastics for stability enhancement and treatment of UVB induced damaged skin. Drug Deliv 28:445–453. doi:10.1080/10717544.2021.1886377 PMC7909477 33620008

[32] He A-Q, Wang L, Wang Q, Luan W-J, Qi F-Z. 2020. Protective effects of micronized fat against ultraviolet B–induced photoaging. Plast Reconstr Surg 145:712–720. doi:10.1097/PRS.0000000000006607 32097312

[33] Wolf P, Weger W, Patra VK, Gruber-Wackernagel A, Byrne SN. 2016. Desired response to phototherapy vs photoaggravation in psoriasis: what makes the difference? Exp Dermatol 25:937–944. doi:10.1111/exd.13137 27376966

[34] Nghiem DX, Kazimi N, Mitchell DL, Vink AA, Ananthaswamy HN, Kripke ML, Ullrich SE. 2002. Mechanisms underlying the suppression of established immune responses by ultraviolet radiation. J Invest Dermatol 119:600–608. doi:10.1046/j.1523-1747.2002.01845.x 12230501

[35] Lerche CM, Al-Chaer RN, Wulf HC. 2023. Does systemic hydrochlorothiazide increase the risk of developing ultraviolet radiation-induced skin tumours in hairless mice? Exp Dermatol 32:341–347. doi:10.1111/exd.14703 36333872

[36] Leonel C, Sena IFG, Silva WN, Prazeres PHDM, Fernandes GR, Mancha Agresti P, Martins Drumond M, Mintz A, Azevedo VAC, Birbrair A. 2019. *Staphylococcus epidermidis* role in the skin microenvironment. J Cell Mol Med 23:5949–5955. doi:10.1111/jcmm.14415 PMC6714221 31278859

[37] Mohamed HRH, Hemdan SHA, El-Sherif AA. 2025. Y2O3NPs induce selective cytotoxicity, genomic instability, oxidative stress and ROS mediated mitochondrial apoptosis in human epidermoid skin A-431 cancer cells. Sci Rep 15:1543. doi:10.1038/s41598-024-82376-w PMC11718274 39789066

[38] Chen H-Y, Lin C-H, Wu Y, Wang B, Kui M-H, Xu J, Ma H-S, Li J-P, Zeng J-S, Gao W-H, Chen K-F. 2024. Protective effects of degraded *Bletilla striata* polysaccharides against UVB-induced oxidative stress in skin. Int J Biol Macromol 277:134462. doi:10.1016/j.ijbiomac.2024.134462 39098666

[39] Lee E, Ahn H, Park S, Kim G, Kim H, Noh M-G, Kim Y, Yeon J, Park H. 2022. *Staphylococcus epidermidis* WF2R11 suppresses PM2.5-mediated activation of the aryl hydrocarbon receptor in HaCaT keratinocytes. Probiotics & Antimicro Prot 14:915–933. doi:10.1007/s12602-022-09922-8 PMC9474527 35727505

[40] Zhang B-X, Zhao Z-S, Meng X-Y, Chen H-G, Fu G-J, Xie K-L. 2018. Hydrogen ameliorates oxidative stress via PI3K-Akt signaling pathway in UVB-induced HaCaT cells. Int J Mol Med 41:3653–3661. doi:10.3892/ijmm.2018.3550 29532858

[41] Lu X-Y, Kuai L, Huang F, Jiang J-S, Song J-K, Liu Y-Q, Chen S, Mao L-J, Peng W, Luo Y, Li Y-Y, Dong H-Q, Li B, Shi J-L. 2023. Single-atom catalysts-based catalytic ROS clearance for efficient psoriasis treatment and relapse prevention via restoring ESR1. Nat Commun 14:6767. doi:10.1038/s41467-023-42477-y PMC10600197 37880231

[42] Wei M, He X, Liu N, Deng H. 2024. Role of reactive oxygen species in ultraviolet-induced photodamage of the skin. Cell Div 19:1. doi:10.1186/s13008-024-00107-z PMC10787507 38217019

[43] Camillo L, Gironi LC, Zavattaro E, Esposto E, Savoia P. 2022. Nicotinamide attenuates UV-induced stress damage in human primary keratinocytes from cancerization fields. J Invest Dermatol 142:1466–1477. doi:10.1016/j.jid.2021.10.012 34695410

[44] Ding C-B, Peng X-J, Yang J-L, Chen K-C, Liu X-L, Zhao Y-C, Zhang S, Sun S-W, Zhang J-P, Ding Q-T, Liu S, Liu W-C. 2023. Rg3-loaded P407/CS/HA hydrogel inhibits UVB-induced oxidative stress, inflammation and apoptosis in HaCaT cells. Biomedicine & Pharmacotherapy 165:115177. doi:10.1016/j.biopha.2023.115177 37467650

[45] Chen Z-E, Wufuer R, Ji J-H, Li J-F, Cheng Y-F, Dong C-X, Taoerdahong H. 2017. Structural characterization and immunostimulatory activity of polysaccharides from *Brassica rapa* L. J Agric Food Chem 65:9685–9692. doi:10.1021/acs.jafc.7b03902 28994289

[46] Ge Y-C, Li M, Bai S-R, Chen C, Zhang S-L, Cheng J-M, Wang X-B. 2024. Doxercalciferol alleviates UVB-induced HaCaT cell senescence and skin photoaging. Int Immunopharmacol 127:111357. doi:10.1016/j.intimp.2023.111357 38104366

[47] Skabytska Y, Biedermann T. 2016. *Staphylococcus epidermidis* sets things right again. J Invest Dermatol 136:559–560. doi:10.1016/j.jid.2015.11.016 26902125

[48] Charachit N, Sukhamwang A, Dejkriengkraikul P, Yodkeeree S. 2022. Hyperoside and quercitrin in *Houttuynia cordata* extract attenuate UVB-induced human keratinocyte cell damage and oxidative stress via modulation of MAPKs and Akt signaling pathway. Antioxidants (Basel) 11:221. doi:10.3390/antiox11020221 PMC8868276 35204104

[49] Huang W-C, Huang M-C, Ouyang H, Peng J-W, Liang J. 2018. Oridonin inhibits vascular inflammation by blocking NF-κB and MAPK activation. Eur J Pharmacol 826:133–139. doi:10.1016/j.ejphar.2018.02.044 29518395

[50] Zheng Q-W, Yang SJ, Yi EJ, Park SJ, Jin X-J, Nguyen TTM, Yi G-S, Jeon Y-J, Yi T-H. 2025. Enzyme-assisted *Rosa davurica* mitigates UV-induced skin photodamage by modulating apoptosis through Nrf2/ARE and MAPK/NF-κB pathways. J Photoch Photobio B 263:113098. doi:10.1016/j.jphotobiol.2025.113098 39787977

[51] An Y-N, Zhang H-F, Wang C, Jiao F-T, Xu H-Y, Wang X-F, Luan W-J, Ma F-X, Ni L-H, Tang X-D, Liu M-Y, Guo W-Y, Lu Y. 2019. Activation of ROS/MAPKs/NF-κB/NLRP3 and inhibition of efferocytosis in osteoclast-mediated diabetic osteoporosis. FASEB J 33:12515. doi:10.1096/fj.201802805RR PMC6902677 31461386

[52] Shi X-Y, Li M, Huang Q-M, Xie L-M, Huang Z-B. 2023. Monacolin K induces apoptosis of human glioma U251 cells by triggering ROS-mediated oxidative damage and regulating MAPKs and NF-κB pathways. ACS Chem Neurosci 14:1331–1341. doi:10.1021/acschemneuro.3c00104 36917811

[53] Vicanolo T, Özcan A, Li JL, Huerta-López C, Ballesteros I, Rubio-Ponce A, Dumitru AC, Nicolás-Ávila JÁ, Molina-Moreno M, Reyes-Gutierrez P, et al.. 2025. Matrix-producing neutrophils populate and shield the skin. Nature 641:740–748. doi:10.1038/s41586-025-08741-5 PMC12074881 40108463

[54] Wang Z-Y, Chen H-X, Wang Y-X, Wu C-N, Ye T, Xia H, Huang R-F, Deng J-X, Li Z-Y, Huang Y-D, Yang Y. 2024. Recombinant filaggrin-2 improves skin barrier function and attenuates ultraviolet B (UVB) irradiation-induced epidermal barrier disruption. Int J Biol Macromol 281:136064. doi:10.1016/j.ijbiomac.2024.136064 39341309

[55] Vats K, Kruglov O, Mizes A, Samovich SN, Amoscato AA, Tyurin VA, Tyurina YLY, Kagan VE, Bunimovich YL. 2021. Keratinocyte death by ferroptosis initiates skin inflammation after UVB exposure. Redox Biol 47:102143. doi:10.1016/j.redox.2021.102143 PMC8487085 34592565

[56] Ito T, Seo N, Yagita H, Tsujimura K, Takigawa M, Tokura Y. 2003. Alterations of immune functions in barrier disrupted skin by UVB irradiation. J Dermatol Sci 33:151–159. doi:10.1016/s0923-1811(03)00177-4 14643520

[57] Rinaldi AO, Li M, Barletta E, D’Avino P, Yazici D, Pat Y, Ward S, Burla D, Tan G, Askary N, Larsson R, Bost J, Babayev H, Dhir R, Gaudenzio N, Akdis M, Nadeau K, Akdis CA, Mitamura Y. 2024. Household laundry detergents disrupt barrier integrity and induce inflammation in mouse and human skin. Allergy 79:128–141. doi:10.1111/all.15891 37766519

[58] Zheng Y, Hunt RL, Villaruz AE, Fisher EL, Liu R, Liu Q, Cheung GYC, Li M, Otto M. 2022. Commensal *Staphylococcus epidermidis* contributes to skin barrier homeostasis by generating protective ceramides. Cell Host Microbe 30:301–313. doi:10.1016/j.chom.2022.01.004 PMC8917079 35123653

[59] Yao Y, Cai X-Y, Fei W-D, Ye Y-Q, Zhao M-D, Zheng C-H. 2022. The role of short-chain fatty acids in immunity, inflammation and metabolism. Crit Rev Food Sci Nutr 62:1–12. doi:10.1080/10408398.2020.1854675 33261516

[60] Glatthardt T, Campos JC de M, Chamon RC, de Sá Coimbra TF, Rocha G de A, de Melo MAF, Parente TE, Lobo LA, Antunes LCM, Dos Santos KRN, Ferreira RBR. 2020. Small molecules produced by commensal *Staphylococcus epidermidis* disrupt formation of biofilms by *Staphylococcus aureus*. Appl Environ Microbiol 86:e02539-19. doi:10.1128/AEM.02539-19 PMC7028967 31862721

[61] Huang T-Y, Jiang Y-E, Scott DA. 2022. Culturable bacteria in the entire acne lesion and short-chain fatty acid metabolites of *Cutibacterium acnes* and *Staphylococcus epidermidis* isolates. Biochem Biophys Res Commun 622:45–49. doi:10.1016/j.bbrc.2022.06.068 35843093

[62] Geng W-L, Zhang Y, Cao Y-H. 2025. Bacteriostasis and anti-Inflammation of *Staphylococcus epidermidis* fermentation broth to propionibacterium acnes. Cosmetics 12:33. doi:10.3390/cosmetics12020033

[63] Shao L, Li T, Yang S-Z, Ma L-J, Cai B-R, Jia Q-W, Jiang H, Bai T-M, Li Y. 2025. The prebiotic effects of fructooligosaccharides enhance the growth characteristics of *Staphylococcus epidermidis* and enhance the inhibition of *Staphylococcus aureus* biofilm formation. Intern J of Cosmetic Sci 47:155–167. doi:10.1111/ics.13020 39246292

